# Anthropogenic disturbance has altered the habitat of two Azorean endemic coastal plants

**DOI:** 10.1186/s12862-024-02300-8

**Published:** 2024-08-20

**Authors:** Rúben M. Correia Rego, Mónica Moura, Maria Olangua-Corral, Guilherme Roxo, Roberto Resendes, Luís Silva

**Affiliations:** 1 CIBIO, Centro de Investigação em Biodiversidade e Recursos Genéticos, Faculdade de Ciências e Tecnologias, InBIO Laboratório Associado, Universidade dos Açores, R. Mãe de Deus 13A, Ponta Delgada, 9500-321 Portugal; 2 BIOPOLIS Program in Genomics, Biodiversity and Land Planning, CIBIO de Vairão, Vairão, Portugal; 3 Faculdade de Ciências e Tecnologias, UNESCO Chair – Land Within Sea: Biodiversity & Sustainability in Atlantic Islands, Universidade dos Açores, R. Mãe de Deus 13A, Ponta Delgada, 9500-321 Portugal; 4 Banco Germoplasma & Dpto. Biología Reproductiva, Jardín Botánico Canario “Viera y Clavijo” – u.a. CSIC, Las Palmas de Gran Canaria, Spain

**Keywords:** Anthropogenic disturbance, Coastal degradation, Invasive species, Conservation, Coastal plant communities, *Campanula Vidalii*, *Lotus Azoricus*

## Abstract

**Background:**

Anthropogenic threats are causing alteration of coastal areas worldwide. Most of the coastal biodiversity is endangered, taking a particular toll on island ecosystems, like the Azores. To better understand the biotic and abiotic factors constraining the distribution and conservation status of two endemic plants, *Azorina vidalii* (Campanulaceae) and *Lotus azoricus* (Fabaceae), we performed a global survey of coastal plant communities in the archipelago, also covering environmental descriptors, natural and anthropogenic threats. Moreover, we revised their IUCN conservation status and estimated the population fractions within protected areas.

**Results:**

Non-indigenous plants were commonly found in plots with or without the target endemics, contributing to the absence of well-defined coastal plant communities. Nonetheless, indigenous taxa commonly occurred at the plots with *L. azoricus*. With a larger area of occurrence, *A. vidalii* ecological niche differed from that of *L. azoricus*, the latter being restricted to dry and rocky sea cliffs, mostly in Santa Maria Island. Besides the presence of invasive plants, signs of habitat destruction, trampling and grazing, and of natural threats, such as coastal erosion, were commonly observed.

**Conclusions:**

Occurrence data indicated an endangered status for both species, although this would change to critically endangered for *L. azoricus* when using smaller-sized occurrence cells. Both species are threatened since their habitat is restricted to a very narrow vegetation belt, strongly limited by sea influence and human pressure, and with the frequent presence of invasive plants. While focusing on two endemic plants, our study allowed a broader view of the impact of anthropogenic disturbance on Azorean coastal plant communities.

**Supplementary Information:**

The online version contains supplementary material available at 10.1186/s12862-024-02300-8.

## Introduction

Coastal areas function as the connection zone between land, sea, and atmosphere, and are subjected to high levels of oscillation, making them particularly sensitive to natural and anthropogenic disturbance [1].

In oceanic islands, intensification of traditional land uses [2, 3] and the growing expansion of human activities into the coastal areas originate habitat loss, changes in vegetation structure and the fragmentation of endemic plant populations [4], leading to biodiversity loss, plant extinctions [5, 6] and to decreases in abundance and diversity [7]. Among these threats, the proliferation of non-indigenous taxa [8], biological invasions [4, 7], and climate change are paramount. The latter is expected to increase the frequency of extreme weather events, to cause sea level rise, and accelerate coastal erosion [9, 10] further altering coastal plant populations in oceanic islands [1, 9]. Moreover, many populations of indigenous taxa are often found outside the circumscription of protected areas, making their conservation more difficult, due to lower land use restrictions and monitoring [11].

The Azores Archipelago integrates the Macaronesian biodiversity hotspot, being characterised by a 7% rate of endemic vascular plants [12], translating to approximately 97 taxa [13], but with more than 3000 introduced plant taxa [14]. Most species have been introduced after the Portuguese settlement, as described in historic literature [15]. Anthropogenic change included the installation of agricultural crops, orchards, vineyards and hedgerows, and the introduction of farm animals and other terrestrial vertebrates that became established in the wild.

More recently, other threats gained relevance, such as the expansion of pasturelands into the coast, the construction of infrastructure, the occasional deposition of solid waste at coastal areas, and the cultivation and subsequent spread of alien species, becoming large-scale invaders [15, 16]. Furthermore, marine erosion of coastal areas is a well-known phenomenon in different types of sand and boulder beaches in the Azores [17]. These events narrowed the occurrence of endemic vegetation to relatively inaccessible areas, such as mountain slopes, craters, or coastal cliffs [16, 18]. Therefore, there is an urgent need to preserve the natural heritage of the Azores [19], that is, designing holistic recovery plans for endangered plants, focussing on monitoring, ecological modelling, habitat restoration and genetics [20].

Among Azorean threatened plant taxa, coastal endemic withstands considerable levels of natural and anthropogenic disturbance [19]. Recent projects addressed reproductive and morphological traits of several Macaronesian endemics (MacFlor: INTERREG MAC 2014–2020 MAC/4.6d/190; MacFlor 2: MAC2/4.6d/386). Project Life Vidalia specifically aimed to improve the conservation status of *Azorina vidalii* H.C.Watson (Campanulaceae), and *Lotus azoricus* P.W.Ball (Fabaceae), through population reinforcement, and habitat restoration, in the islands of Faial, São Jorge and Pico (see https://www.lifevidalia.eu/). Both endemic taxa have been considered as top priorities for conservation in the Azores, being protected by Azorean legislation (Decreto Legislativo Regional n.o. 15/2012/A, Anexo II), by the Natura 2000 Network, under the EU Habitats Directive (Council Directive 92/43/CEE of 21 May 1992), and by Berne Convention [19]. Previous studies suggest that some populations may be declining or even disappearing [21, 22].

According to literature [23–27], coastal plant communities include: (i) Coastal scrubland; (ii) Chamaephyte plant communities from rocky coasts (e.g., rolled pebbled beaches); (iii) Halophyte and halohydrophyte meadows; (iv) Vegetation typical from sandy beaches or dunes; (v) Coastal wetlands (e.g., halophyte reeds); (vi) Soggy meadows, and coastal brackish water ponds. A global quantitative assessment of the coastal plant communities is currently pertinent, given the emergence of several anthropogenic threats, the conservation projects in place, and the present network of protected areas. Thus, herein we have focused on two different taxa, one with a broad and another with a narrow occurrence area, also considering the factors potentially conditioning their ecological niches.

Within this framework we formulated two sets of research questions:

- The first regarding the definition of their present habitat - are there significant differences in terms of: (i) plant community and (ii) environmental descriptors between areas with or without the two taxa?
- The second regarding habitat change and conservation status: (i) Are coastal plant communities still dominated by indigenous taxa? (ii) Are there any relevant threats present at the occurrence areas? (iii) Are the occurrences mainly found within protected areas? and (iv) Has their conservation status improved in recent years?

Based on the framework described above, as our starting hypotheses, (i) we expect that non-indigenous plant taxa presently correspond to a relevant component of the herbaceous coastal plant communities; (ii) we expect that the coastal habitat is under natural and anthropogenic threats; and (iii) we don’t expect a deterioration of their conservation status, given the areas designated for conservation and the restoration measures being implemented.

We performed a thorough ecological survey of the herbaceous coastal plant communities in the nine Azores islands: (i) we included a comparison of the plant communities where the target taxa were present or absent, considering the effect of anthropogenic threats like invasive species and changes in the vegetation structure; (ii) we analysed environmental descriptors (climate, altitude, substrate) to better define their habitats; (iii) we identified potential threats; and (iv) we applied the IUCN criteria to revise their conservation status, while also evaluating the effectiveness of protected areas in covering the respective populations. Although our main targets were the two endemic taxa − *Azorina vidalii* and *Lotus azoricus*−, we consider the global analysis of the herbaceous coastal communities as a baseline requirement for a holistic understanding of their habitats and conservation status.

## Methods

### Study site

**Fig. 1:**
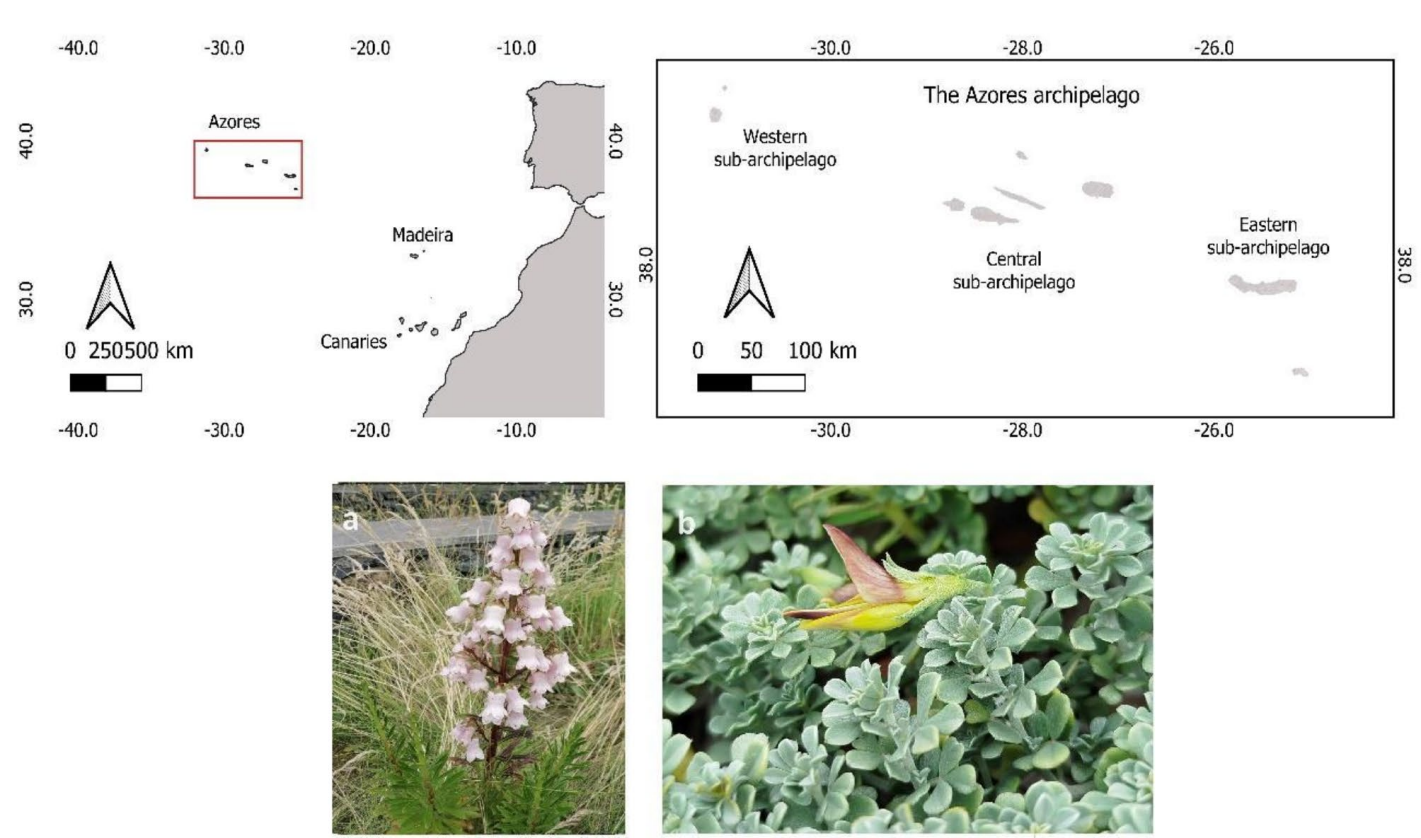
Geographical location of the Azores archipelago, the islands composing each subarchipelago of the Azores and target species: (**a**) *Azorina vidalii*; (**b**) *Lotus azoricus*

The Azores archipelago (37º-40º N, 25–31 W; Fig. 1) is situated in the north Atlantic Ocean, and it’s composed of nine volcanic islands and several islets, divided into three groups: Western (Flores and Corvo), Central (Terceira, Graciosa, São Jorge, Pico, and Faial) and Eastern (São Miguel and Santa Maria). The archipelago is in a warm temperate zone with high relative humidity, low thermal amplitude, and rainfall throughout the year. The average temperature at the coastal areas ranges between 14º and 17ºC [28].

### Target species

*Azorina vidalii* is a synonym of *Campanula vidalii* (H.C.Watson) Feer. Its taxonomy is currently under revision, due to clustering within other *Campanula* species in a published phylogeny [29], is a glabrous chamaephyte common below 50 m a.s.l., found in all islands [12], rarely occurring at altitudes above 100 m [19]. The phytosociological alliance *Euphorbio azoricae*-*Festucion petraeae* [27], included the association *Azorinetum vidalii* [23], characteristic of sea cliff communities. However, this species displays a very diverse ecology, being associated with different coastal plant communities, on different islands, from common rocky chamaephyte communities to halohydrophyte meadows, typical of Corvo and Terceira islands [24].

The endemic legume, *Lotus azoricus*, is a semi-herbaceous hemicryptophyte, whose distribution has been referred to the islands of Santa Maria, São Miguel, Pico, São Jorge and Flores [12], however, its presence in some islands (e.g. São Miguel and Flores) lacks confirmation. It grows on rocky shores, coastal cliffs and lava flows from 5 up to 95 m a.s.l., on sand or rubble soils from incipient to a few centimetres thick [19, 21]. *Lotus azoricus* has been associated with the phytosociological alliance of *Tolpido succulentae*-*Agrostion congestiflorae* [25]. Although *L. azoricus* has been considered as a synonym of *L. argyrodes* R.P.Murray [30], a divergence time of 2.5 Mya between the two species [31] has been estimated, and available DNA sequences show differences at several positions. Therefore, as is often the case with plant taxa isolated in different archipelagos [32], we consider the two taxa as separate evolutionary, ecological and taxonomic units.

### Distribution of sampling stations

Using Quantum GIS 3.28.2 [33], the coastal area of each island was divided using a grid of 500 × 500 m, allowing to overview which areas would be accessible for sampling, as well as to make sampling distributed homogeneously across the coast. All locations were georeferenced using a GPS device (Garmin Montana, 680). In total, we selected 148 sampling stations (500 × 500 m cells) across the nine islands of the archipelago that were accessible by walking trails. These sampling stations were selected before the start of field work, independently of the distribution of the two target plants, and previously to any field observation.

### Plant community sampling

Field work was carried out from June to November of 2022. Following previous work [34], we used 5 × 5 m plots to prospect and describe coastal plant communities. We used an average of three plots at each sampling station, with a minimum of one plot at most sampling stations without the target species, and a maximum of eleven plots at a sampling station in Santa Maria Island with a relatively large extension and elevation span.

In total 231 5 × 5 m plots were sampled, including plots with and without the target plants. Therefore, four plot types were defined: (i) A – plant communities with *Azorina vidalli* only; (ii) L – plant communities with *Lotus azoricus* only; (iii) B – plant communities including both target species; and C – plant communities where both species were absent.

In many locations, the area available for the target herbaceous coastal plant communities was constrained by the sea level, below, and by dense scrubland with indigenous or/and non-indigenous taxa, or humanised areas (housing, crops, pastures), above. We focused our sampling effort on that intermediate belt. In some cases, like coastal cliffs with smoother slopes, the herbaceous coastal vegetation extended to relatively high elevations (100 m).

At each 5 × 5 m plot we recorded the percent cover for each vascular plant taxa, which was visually estimated by a vertical projection of above ground plant parts at each of four equal sized subplots. Plant species were identified in situ or sampled and later identified in the laboratory with the help of field guides and floras [18, 35–37] (Plant material identification undertaken by Luís Silva, Guilherme Roxo and Rúben M. C. Rego). The sampled voucher specimens were preserved in collection at the AZB herbarium (Voucher ID’s AZB 4311 to AZB 4381; Azores University, Ponta Delgada, Portugal). In total, 197 taxa were recorded in the 231 plots made (Supplementary Table S1).

### Colonisation status

To evaluate the level of anthropogenic alteration in the composition of the plant communities, we categorised the plant taxa according to their colonisation status, following species lists [12, 38] and local legislation (DLR n.º 15/2012/A, 2 de Abril), into (see Supplementary Table S2): indigenous (i.e., taxa that arrived or evolved on the islands in the absence of human intervention), with two subgroups – “native” and “endemic”; (ii) non-indigenous (i.e., taxa that were intentionally or accidentally introduced as the result of human activities), with two subgroups – “naturalised”, and “invasive”.

### Life-forms

To evaluate possible differences in vegetation structure between plots with or without the target species, we used the Raunkiaer [39] life-form system, revised by Braun-Blanquet [40], to categorise plant taxa, based on the position of the resting buds, considering the following types (see Supplementary Table S2): therophyte, hemicryptophyte, chamaephyte, geophyte, and phanerophyte.

### Habitat ecology

We used published floras [35, 36, 41] to categorise species according to the ecology of their habitats, into: xerophyte, halophyte, mesophyte, hygrophyte and generalist (see Supplementary Table S2).

### Environmental descriptors evaluated in situ

We used the following environmental descriptors to describe the habitat of the target species: elevation, slope, exposure, type of substrate and threats to the habitat. Exposure and elevation were recorded using a portable GPS (Garmin Montana, 680).

#### Substrate

The following substrates were considered and defined, following literature [42] (see Supplementary Table S2): sand, clay, *lapilli*, lava flow, boulders, rolled pebbles, soil and rocky soil.

#### Soil parameters

Soil was collected whenever it was present, since in most plots we only found a rocky substrate, without a soil layer. The 50 samples were sent to the Soil and Plant Laboratory of the University of Trás-os-Montes e Alto Douro (Vila Real, Portugal) for the following analyses: pH (H_2_O and KCl), organic matter (OM), extractable content of the main nutrients (phosphorus and potassium, by the Eletrolite Replacement and Olsen method), cations (Ca^++^, Mg^++^, K^+^, Na^+^, Al_3_^++^), effective cation exchange capacity (CEC), electrical conductivity (1:5 soil to water ratio), total nitrogen and texture (granulometry).

#### Identified threats

To define the most common potential threats to the survival of the target species, we recorded signs of the effect of natural or anthropogenic threats in situ. Natural threats included (see Supplementary Table S2): (i) coastal erosion; (ii) direct sea submersion; and (iii) storms. Anthropogenic threats included: (i) agriculture; (ii) animal husbandry; (iii) construction work; (iv) waste disposal; (v) habitat destruction; (vi) human infrastructure; (vii) trampling; and (viii) invasive species.

### Climatic data

We used climatic data to determine if there were relevant variations or differences between the different plot types, regarding the main bioclimatic descriptors. Climate data was obtained through the CHELSA (Climatologies at high resolution for the earth’s land surface) V.2.1 database [43]. We used 19 bioclimatic variables (11 related with temperature and 8 with precipitation), consisting of data retrieved between 1981 and 2010. A full list of variables is provided in Supplementary Table S3.

### Statistical treatment

#### Plant diversity

To compare diversity levels between plot types, we calculated species richness (number of taxa per plot), Shannon entropy (total diversity per plot), maximum entropy [44] (maximum theoretical diversity) and evenness [45].

#### Quantitative variables

For quantitative variables, i.e., diversity indexes, quantitative environmental descriptors, comparison of plot types was performed using boxplots and the Kruskal-Wallis test, followed by non-parametric multiple comparison tests (R “pgirmess” package [46]). We opted for a conservative approach, using non-parametric tests, since we could not ensure normality and homoscedasticity, required for parametric tests.

#### Categorical variables

For categorical variables, i.e., frequency of threats and substrate, we used Pearson’s chi-square test followed by a test for comparison of proportions, included in the package “gmodels” [47], and bar charts for graphical representation. For soil texture, due to the small sample size, we used Fisher’s exact test and the chi-square test with the option of bootstrap, to confirm possible significant differences.

#### Statistical applications and output

All the statistical analyses were performed using Rx64 4.2.3 [48]. Overall results from the Kruskal-Wallis and Pearson’s chi-square tests are given in Supplementary Tables S4, S5, S6, S7, S8, S10 and S12, and the significant differences between plot types are indicated using different letters in the respective figures. We should note that the Kruskal-Wallis test statistic, H, is provided as a chi-squared approximation in the R output, as is common in many statistical applications.

#### Species frequency, cover, and importance

In order to determine the plant taxa that were physiognomically dominant, we calculated and plotted the frequency, abundance, and importance of each taxon as follows [49]: (i) frequency as the percentage of plots with the taxon; (ii) abundance (based on percent cover) as the total abundance of the taxon, divided by the total abundance of all taxa; and (iii) importance as frequency (%) + abundance (%) divided by two.

#### Clustering and ordination

To detect possible differences between plot types, we used hierarchical cluster analysis with the “vegan” package [50], based on species cover. Several combinations of distance and agglomeration methods [51] were considered and ultimately, we concluded that Bray-Curtis dissimilarity combined with Unweighted Pair Group Mean Average (UPGMA) provided the highest cophenetic correlation coefficient. The optimal number of plot groups in the dendrogram was determined using two algorithms: (i) according to silhouette widths (Rousseeuw quality index) [52], and (ii) according to the Mantel statistic (Pearson) [53]. We also represented the Bray-Curtis dissimilarity matrix using Non-Metric Multidimensional Scaling (NMDS), as commonly used in numerical ecology.

#### Indicator species analysis

To detect possible differences between plot types, we used indicator species analysis with the R package “Indicspecies” [54], consisting in an improvement of the IndVal method initially established by Dufréne and Legendre [55]. As an abundance metric, species percent cover was used. The algorithm calculates fidelity (limitation to a single site or set of sites) and consistency (consistent species occurrence among sites within site groups) and returns a statistic (IndVal) and the corresponding *p*-value.

#### Binary logistic regression

Following previous work [56], we used the “glm()” function in R to calculate binomial generalised linear models, to determine which factors (i.e., climate, substrate, percent of endemic, native, naturalised and invasive taxa, or a mix of several factors) potentially affect the occurrence of the target species in the coastal habitats.

For both species, a null model, not including explanatory variables, but only an intercept or model constant, was considered as a benchmark, for comparison with the explanatory models described below. Models underperforming when compared with the null model were excluded.

We tested several models, including different combinations of the explanatory variables: (i) a bioclimatic model including the principal components extracted from a principal component analysis, applied to the 19 bioclimatic variables; (ii) a model assessing the effect of substrate types; (iii) a model regarding the contribution of plants with different colonisation status, and diversity measures; (iv) a saturated model including the effect of all previous factors; and (v) several models resulting from the simplification of the saturated model, converging to a simplified final model.

The maximum likelihood approach was used for model selection and simplification, based on Akaike’s Information Criterion (AIC) - the lower the better. All models were compared with a null model only including an intercept. Given the large number of samples (more than 200 observations) and the fact that the possible outcome is binary (the species is present or absent), we consider the application of binomial GLMs as appropriate, following previous work [56]. We also performed a final model selection, by keeping only those variables that would exhibit significant regression coefficients.

### Populations included in Natura 2000 or Island Natural Parks

To access whether the populations of the target species were located within the areas covered by the Natura 2000 network or by Island Natural Parks in the Azores, the georeferenced populations of *A. vidalii* and *L. azoricus* were mapped in QGiS [33] and intersected with the shapefiles representing the protected areas (Source: Azorean Government).

### IUCN Red List assessment of the target species

We evaluated the conservation status of the target species following the guidelines of the IUCN Red List, v.15.1 [57]. We performed calculations of the extent of occurrence (EOO) and of the area of occupancy (AOO) using GeoCAT [58]. We calculated AOO using 2 × 2 km grid cells (area of 4 km [2]) and based the estimates of the number of mature individuals on counts made during field work.

## Results

### Plant community sampling

A total of 197 taxa were recorded, with an average of 9.3 taxa per plot. The highest relative cover and frequency was obtained for the endemic *Festuca petraea* (C: 14.0%, F: 119 occurrences, 51.5%). *Azorina vidalii* (C: 6.5%, F: 99 occurrences, 42.9%) was clearly more abundant and frequent than *Lotus azoricus* (C: 1.4%, F: 17 occurrences, 7.4%). Some native (e.g., *Crithmum maritimum*) and several invasive taxa appeared frequently in the sampled plots (e.g., *Carpobrotus edulis*,* Tetragonia tetragonoides*,* Paspalum dilatatum*,* Arundo donax*,* Cynodon dactylon*), which contributed with high percentages of cover and frequency (Supplementary Figures S1 and S2).

### Colonisation status

**Fig. 2:**
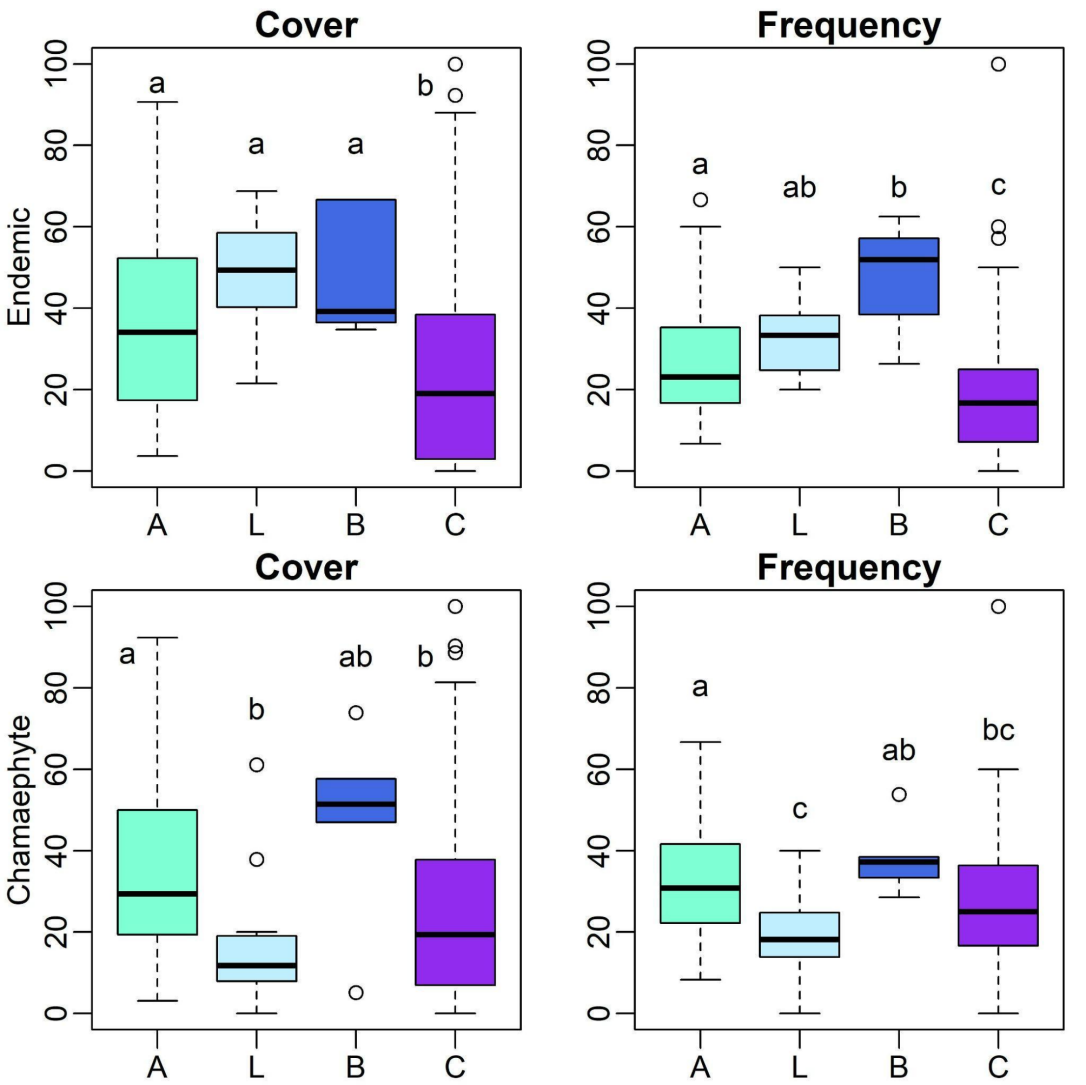
Cover and frequency results for top) endemic plant taxa and bottom) chamaephyte taxa found in 231 plots sampled in coastal areas in the nine Azores islands. Plot types: A – including *Azorina vidalii*, L – including *Lotus azoricus*, B – including both species, C – controls without both species. Different letters indicate significant differences (*p* < 0.05); Results of a non-parametric multiple comparison test applied after Kruskal-Wallis test

The 197 records corresponded to 108 naturalised, 40 invasive, 27 native and 22 endemic taxa. For endemic plant cover, significant differences (*p* < 0.005) were found between plot types A, B and L towards C plots, with lower values for the latter. Endemic plant frequency also showed significant differences between plot types A towards B and C, and between plots L and B, towards plots C, with the lowest values for the latter (Fig. 2; see Supplementary Table S4). No significant differences between plot types were found for the remaining colonisation status, in cover or frequency, with median values around 20% for native and naturalised taxa, and below 20% for invasive taxa.

### Life-forms

We detected significant differences between plots A and plot types L and C for the cover of chamaephytes, with higher values for A and B (Fig. 2). Similarly, we found significant differences in chamaephyte frequency, between plots A and plots L and C, and between plots B and L, with higher values for A and B (Fig. 2, Supplementary Table S5). No significant differences between plot types were found for the cover and frequency of the remaining life forms, with median values ranging 1% for geophytes, below 20% for phanerophytes, and ranging from 20 to 50% for therophytes and hemicryptophytes (data not shown).

### Habitat ecology

Across the ecology types considered, only halophyte, generalist and mesophyte species were relevant, as the cover and frequency of hygrophytes and xerophytes was always below 5% and sometimes null, among plot types. The cover and frequency of mesophytes was around 20%, among plot types. The cover of halophyte species ranged between 70% in L plots and 90% in A and B plots, while the frequency was around 60–70%, among plot types. Generalist species dominated the cover and frequency in C plots (> 40%), appearing also with high cover and frequency in A plots (40%), but much lower in plots with *L. azoricus* (around 25%). No significant differences were detected for the cover and frequency of the ecology types, among plot types, after the Kruskal-Wallis analysis (Supplementary Figure S3; see Supplementary Table S6).

### Plant community clustering

The highest cophenetic correlation was obtained for Bray-Curtis’s dissimilarity Index and UPGMA (0.707). The best value for the number of plot clusters was 32. We found significant differences for Bray-Curtis dissimilarity (Supplementary Table S7) between plot types A and L and C plots, the latter showing the highest values (Supplementary Figure S4).

**Fig. 3:**
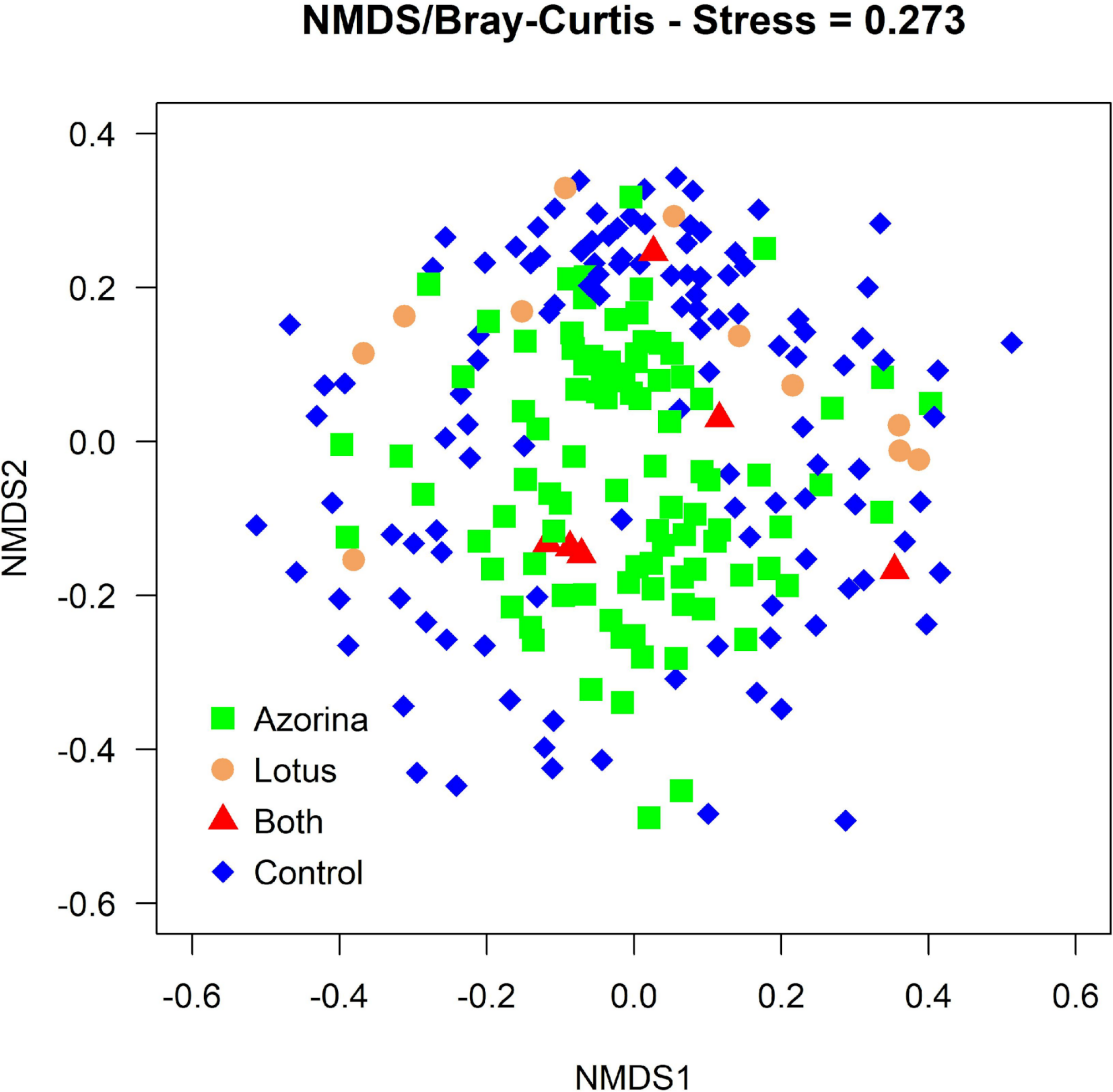
Results of a Non-Metric Multidimensional Scaling (NMDS) applied to the Bray-Curtis dissimilarity matrix, based on 231 plots sampled in coastal areas in the nine Azores islands. Different colours represent the four plot types

The results of the NMDS plot (Fig. 3) showed no clustering of the four plot types. Type C plots were mostly found on the periphery of the plot, while most A plots were concentrated at the middle, and L plots were mostly scattered at the top.

### Plant diversity

**Fig. 4:**
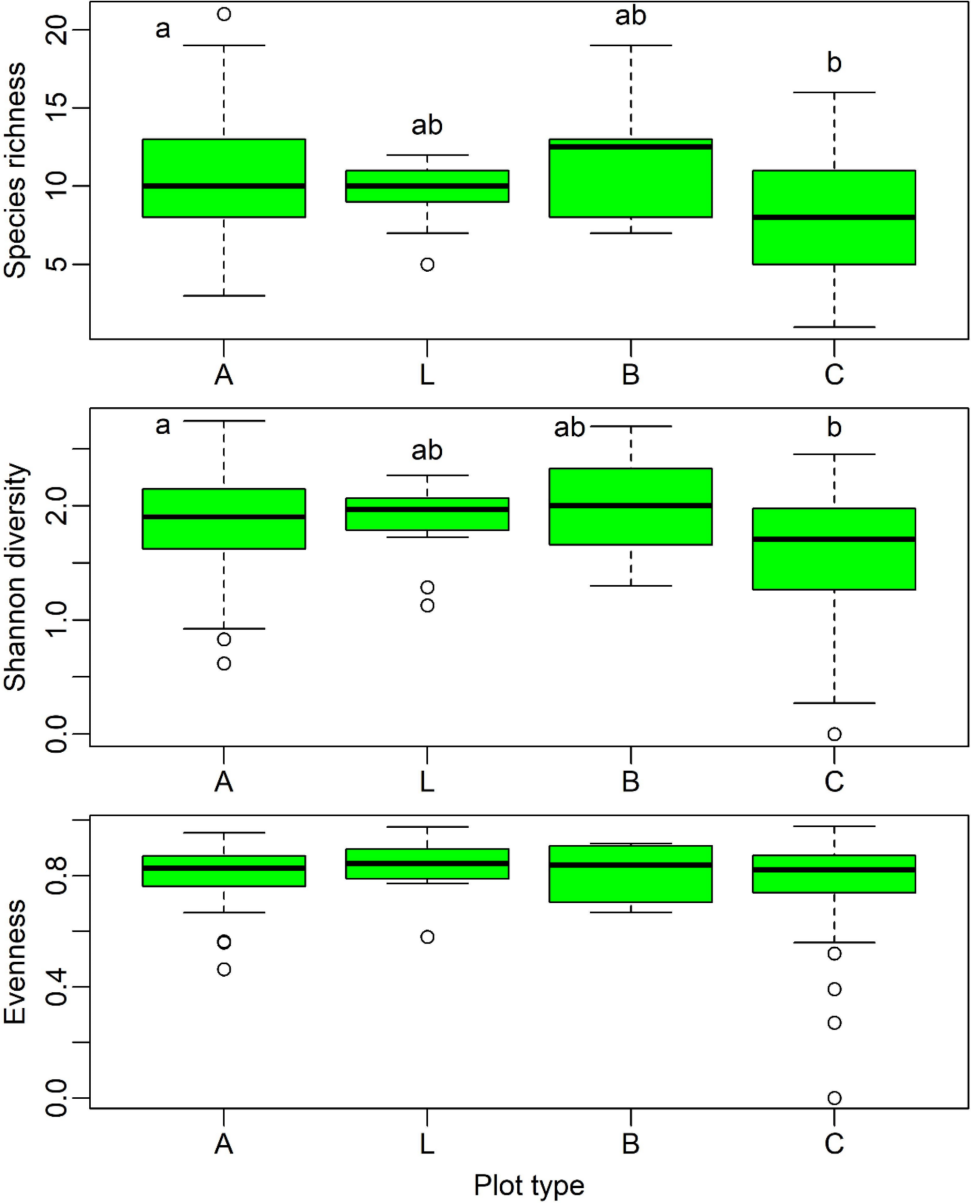
Boxplots of Species richness and Shannon diversity, based on 231 plots sampled in coastal areas in the nine Azores islands. Plot types: A – including *Azorina vidalii*, L – including *Lotus azoricus*, B – including both species, C – controls without both species. Different letters indicate significant differences (*p* < 0.05); Results of a multiple comparison test applied after the Kruskal-Wallis test

We detected significant differences between plot types A and C for species richness and Shannon diversity (Supplementary Table S7), with higher values for the former (Fig. 4), but no significant differences between plot types for evenness which was concentrated around 0.8 (data not shown).

### Indicator species

Considering the four types of plots, we found 14 taxa with significant indicator value (*p* < 0.05), six taxa being associated with one plot type, 7 taxa associated with two plot types, and only one taxon associated with three plot types (Table 1).

**Table 1:** Indicator species associated with four plot types, from 197 taxa retrieved from 231 coastal plots

Plot type	Taxa	Indicator value	*p*-value
L	*Lolium rigidum*	0.587	0.005
	*Agave americana*	0.485	0.020
B	*Spergularia azorica*	0.788	0.005
	*Calendula suffruticosa*	0.707	0.005
	*Gaudinia coarctata*	0.626	0.005
	*Calluna vulgaris*	0.408	0.020
A + B	*Azorina vidalii*	1.000	0.005
L + B	*Lotus azoricus*	1.000	0.005
	*Euphorbia azorica*	0.631	0.035
	*Reichardia picroides*	0.608	0.005
	*Limonium diasii*	0.553	0.015
	*Lysimachia arvensis*	0.473	0.040
	*Plantago lanceolata*	0.427	0.045
A + L + B	*Sonchus tenerrimus*	0.710	0.010

Plot type abbreviations are as follows: A: *Azorina vidalii* only; L: *Lotus azoricus* only; B: both taxa; C: control (without *A. vidalii* or *L. azoricus*)

### Environmental variables

#### Elevation and exposed soil

Significant differences were found between plot types for elevation, with the highest median value for plot type L (Supplementary Figure S5; Supplementary Table S8). No significant differences were found between plot types, for the proportion of exposed soil, which only had relevance in plots B (≤ 20%, data not shown).

#### Type of substrate

**Fig. 5:**
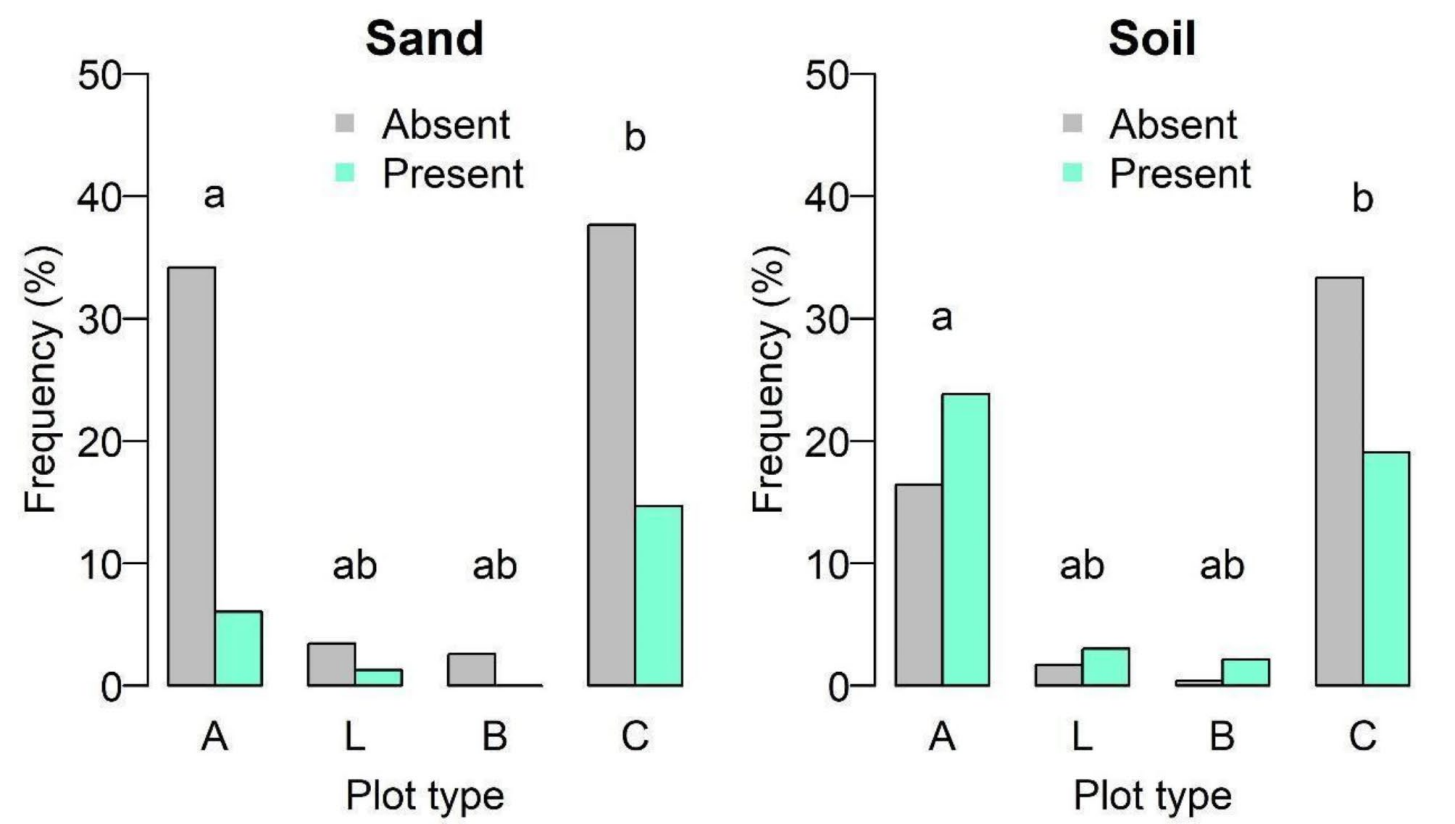
Frequency of sand and soil substrates, based on 231 plots sampled in coastal areas in the nine Azores islands. Plot types: A – including *Azorina vidalii*, L – including *Lotus azoricus*, B – including both species, C – controls without both species. Different letters indicate significant differences (*p* < 0.05); Results of a multiple comparison test applied after chi-square test

Regarding substrate type, six out of nine substrates had a general frequency of at least 10% (*lapilli*, lava flow, boulders, rolled pebbles, sand and soil), but only soil and sand retrieved significant differences in the multiple comparison test, between plot types A and C (Fig. 5; Supplementary Table S8), with a higher frequency of sand in type C plots and a higher frequency of soil in A type plots.

No significant differences were found between plot types, for the remaining substrates, with overall frequencies of 4.8% for clay substrate, 9.5% for rocky soil, 10.8% for rolled pebbles, 22.1% for sand, 24.7% for *lapilli*, 42.8% for lava flow, 48.0% for soil, and the highest frequency, which was of 76.6%, for boulders.

#### Soil parameters

Most soil parameters did not show any significant differences between plot types (Supplementary Table S8). Significant differences were only observed between plots B and C, for the extractable content of phosphorus, with higher levels in the former (Supplementary Figure S6). Although the Kruskal-Wallis test indicated significant differences between the plot types for electric conductivity, the subsequent multiple comparison test failed to confirm those differences.

No significant differences were found between plot types for field texture (Supplementary Table S8). Sand, sandy clay loam and silt loam soil textures obtained overall frequencies below 5%, the frequency of clay loam was 8%, loamy sand obtained 18%, loam had a frequency of 20%, and the highest percentage was observed for sandy loam soils with 46% (Supplementary Figure S7).

#### Climate

The 19 bioclimatic variables were reduced to three principal components, explaining 94% of the variance (Supplementary Figure S9). The first principal component was strongly supported by precipitation variables, while the second principal component showed to be positively influenced by precipitation, and negatively influenced by temperature variables. Finally, the most important variables for the third principal component were related with the mean temperatures in the different quarters of the year, whilst having some mixed influence of both temperature and precipitation variables (Supplementary Table S9). We only found significant differences among plot types for the first two components (Supplementary Table S10), indicating larger precipitation for A type plots, and lower precipitation for plot types L and B.

### Binary logistic regression

In total, 24 models with different explanatory variable combinations were tested, separately, for the two target species (further details on the variables selected for each model are available in Supplementary Table S11).

Based on our best simplified model, with an AICc much lower than the null model, the presence of *A. vidalii* was positively affected by higher species richness and the presence of soil, coupled with higher levels of endemic and naturalised plant cover (Table 2). The occurrence of this species also appears strongly correlated with higher precipitation values, as defined by the first principal component of the climate, and higher temperature and thermal amplitude, as defined by the second principal component.

Based on our best simplified model, with an AICc much lower than the null model, higher values for the frequency of endemic taxa and Shannon diversity appear to be beneficial for the occurrence of *Lotus azoricus* (Table 2). However, it appears negatively affected by the first principal component of the climate, associated with higher precipitation values.

**Table 2:** A Summary of the simplified binary logistic regression models obtained for *Azorina Vidalii* and *Lotus Azoricus*. For the complete set of calculated models see Supplementary Table S10. Explanatory variables defined in the text. Regression coefficients and the respective standard error and significance. AICc for the models and the respective standardised pR^2^ value are also given

Taxon	Explanatory variables	Regression coefficients	Std. Error	Sig.	Model AICc	pR^2^	Null model AICc
*Azorina vidalii*	PCA1 climate	1.839	0.468	***	256.95	0.222	317.50
	PCA2 climate	1.690	0.645	**			
	Species richness	0.182	0.047	***			
	Endemic cover	0.035	0.009	***			
	Naturalised cover	0.020	0.010	*			
	Soil substrate	0.789	0.320	*			
*Lotus azoricus*	PCA1 climate	-3.907	1.038	***	81.55	0.396	132.48
	Shannon Index	2.374	0.854	**			
	Endemic frequency	0.105	0.026	***			

Significance levels are as follows: <0.001 ‘***’ 0.001 ‘**’ 0.01 ‘*’

### Threats

**Fig. 6:**
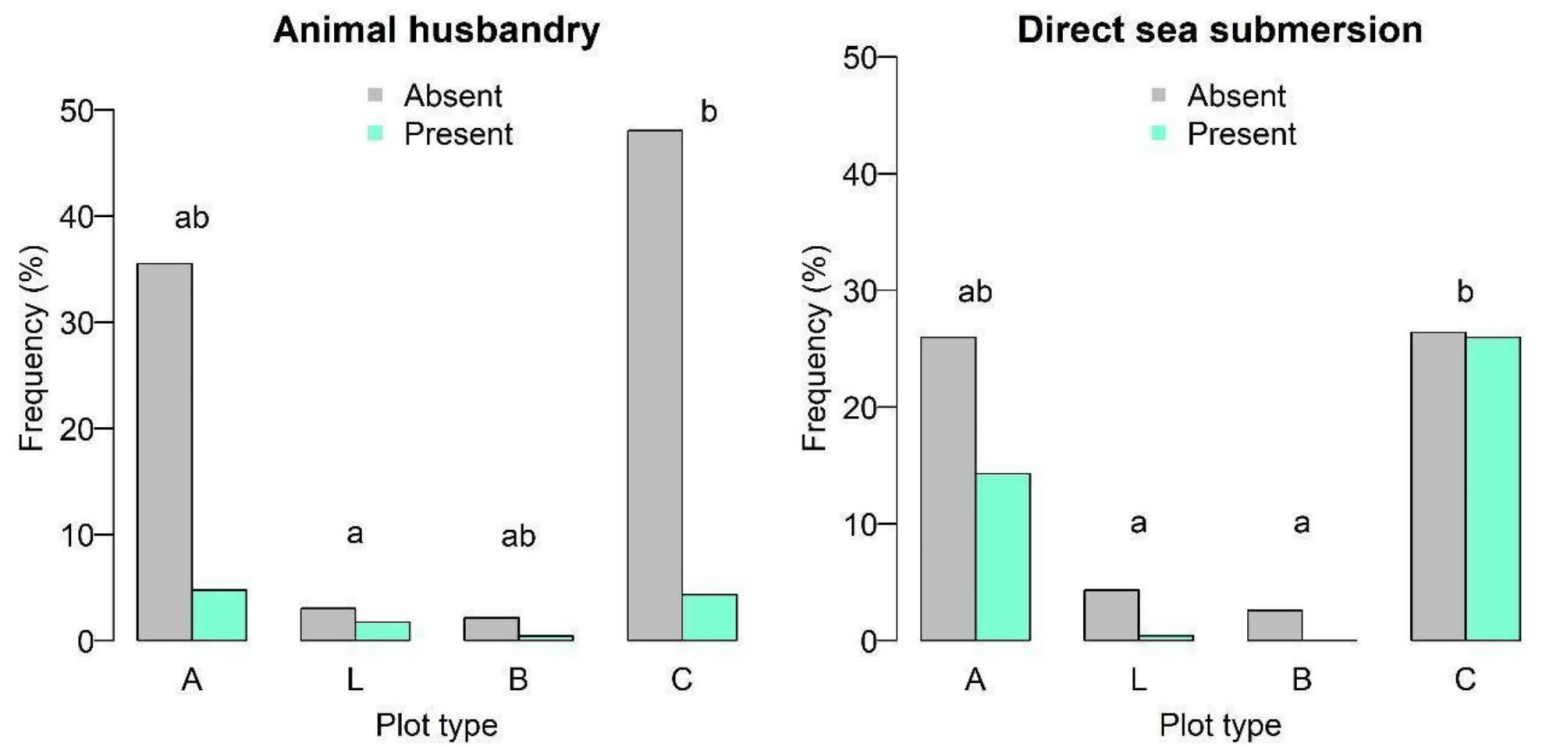
Frequency of animal husbandry and direct sea submersion found in 231 plots made in coastal areas on nine islands of the Azores. Plot types: A – including *Azorina vidalii*, L – including *Lotus azoricus*, B – including both species, C – controls without both species. Different letters indicate significant differences (*p* < 0.05); Results of the multiple comparison test applied after Chi-square test

Nine of the 11 threats considered in this study displayed an overall frequency of at least 10%, including three of natural origin (sea exposure, storms, and coastal erosion), and six anthropogenic (invasive species, trash disposal, human presence, habitat destruction, trampling, and animal husbandry). Figure 6 shows that significant differences were found for direct sea submersion (overall frequency: 40.7%), between plot types L and B, and plots of type C, with a higher frequency in the latter; as well as for animal husbandry (overall frequency: 11.2%), between plots L and C (Supplementary Table S12).

No significant differences were detected, among plot types, for the remaining threats (please see Supplementary Figure S9), obtaining the following overall frequencies: 1.7% for construction work, 9.5% for agriculture, 18.2% for trampling and trails, 38.1% for habitat destruction, 53.7% for coastal erosion, 54.5% for human infrastructure, 58.4% for waste disposals, 71.0% for storms and 82.7% for invasive alien plants.

### Intersection with protected areas

For *A. vidalii*, most plots were located within Protected Areas for Management of Species and Habitats (PAMSH) and Special Conservation Zones (SCZ) (Supplementary Table S13). Most of the INP and Natura 2000 network areas overlapped, except for one site at Pico, which was only covered by an SCZ. The islands of Santa Maria, São Jorge and Flores had the highest percentages of plots within any of the protected frameworks, while in Corvo or Graciosa there was none. Globally, 41.41% of the plots for this species were found within protected areas.

For *L. azoricus*, the highest percentages of plots were also found in PAMSH and SCZ (Supplementary Table S13). All the plots containing this taxon were under protected land in Santa Maria and São Jorge islands, although within different typologies, while only 40% of the plots were within protected areas in Pico. Overall, 82.35% of the plots for this species were found within protected areas.

Although some of the larger islands, such as São Miguel or Pico, contained the greatest amounts of area of occupancy for the two target taxa, it appears that the percentage of populations from these populations under protected areas is lower than in some of the smaller islands (e.g., Santa Maria, Faial, or Flores).

### IUCN evaluation

Table 3 shows the conservation assessments made for the two target species, which resulted in the attribution of the Endangered category to both species.

According to the data available (Supplementary Tables S14 and S15), the evaluation for both species followed conditions B2 and a) since we have observed that populations appear severely fragmented, and b), (iii), due to the observed decrease in the area and quality of the habitat. For *Lotus azoricus*, criteria C2 was also selected, since the populations are small and decline in population size has been observed at some locations, by following a) and the conditions (i) and (ii). However, based on the number of mature individuals, which was 11250, the conservation status of *Azorina vidalii* would be Least Concern, while *Lotus azoricus* would still be in the Endangered category, with only ca. 1520 mature individuals (see Supplementary Table S15). We have also calculated the area of occupancy using 1 × 1 km cells, instead of the IUCN standard of 2 × 2 km, which resulted in the attribution of the Critically Endangered (CR) category to *L. azoricus*.

## Discussion

### Sampling constraints

We have sampled in a systematic way along the coast, wherever access was possible by walking along coastal trails, including habitats such as: coastal cliffs, sand beaches, dunes, and rolled pebbled beaches, streams, lava flows and volcanic gravel.

The coast includes a narrow fringe of coastal vegetation, as described by Tutin [26]. Functioning as an ecotone between marine and terrestrial ecosystems, the zonation from sea level to the upper vegetation belts is determined by the exposure to salinity. Starting from sea level, upwards, we would first find the typical coastal (halophyte) species, such as *Spergularia azorica*, *Crithmum maritimum* and *Euphorbia azorica*, progressing to *Festuca* meadows and, above, to *Erica*-*Morella* scrublands or *Picconia*-*Morella* lowland forest [59]. We focused our sampling effort in the first herbaceous vegetation belts, where the target species generally appear.

However, the presence of inaccessible vertical coastal cliffs, extensive humanised areas (housing, agricultural land, pastures), the occurrence of dense stands of invasive species (e.g., *Arundo donax*, *Pittosporum undulatum*) limited the sampling area available for coastal herbaceous plants, such as our target species.

Nonetheless, we are confident that our sampling scheme was unbiased. We visited all the sites where the target species were previously reported (including recent surveys by LIFE projects), but still, we could not confirm previous records of *L. azoricus* for São Miguel and Flores islands [12].

**Table 3:** Conservation status assessment of *Azorina Vidalii* and *Lotus Azoricus*, following IUCN guidelines

Criteria	Sub-criteria	Condition		Azorina vidalii	Lotus azoricus	Conservation category		
						CR	EN	VU
A Pop. size reduction	A1	Population reduction (measured over the longer of 10 years or 3 generations) based on any of A1 to A4		No data from the previous 10 years or 3 generations, to either observe, estimate, infer or suspect a decrease in population sizes.		≥ 90%	≥ 70%	≥ 50%
	A2, A3, A4					≥ 80%	≥ 50%	≥ 30%
B Geographic range	B1 (Based on EOO)	-		43816.38 km^2^ (LC)	5487.01 km^2^ (VU)	< 100 km^2^	< 5000 km^2^	< 20,000 km^2^
	B2 (Based on AOO)	-		276 km^2^ (EN); 78 km^2^ (EN)*	32 km^2^ (EN); 9 km^2^ (CR)*	< 10 km^2^	< 500 km^2^	< 2000 km^2^
		AND at least 2 of the following 3 conditions						
		(a) Severely fragmented or number of populations		(a)	(a)	1	≤ 5	≤ 10
		(b) (iii) Observed decline in: area, extension and/or quality of the habitats		(b) (iii)	(b) (iii)			
		(c) Extreme fluctuations						
C Small population size and decline	Number of mature individuals	-		11,250 (LC)	1520 (EN)	< 250	< 2500	< 10,000
		AND at least one of C1 or C2						
	C1	An observed, estimated or projected continuing decline of at least (up to a max. of 100 years in future)				25% in 3 yrs./1 gen.	20% in 5 yrs./2 gen.	10% in 10 yrs./3 gen.
	C2	An observed, estimated, projected or inferred continuing decline AND at least 1 of the following 3 conditions:	(a) (i) Number of mat. ind. in each subpopulation	(a) (i) (VU)	(a) (i) (VU)	≤ 50	≤ 250	≤ 1000
			(a) (ii) % of mat. ind. in one subpopulation =		(a) (ii) (CR)	90–100%	95–100%	100%
			(b) Extreme fluctuations in the number of mat. ind.					
D Very small or restricted population	D1 Number of mature individuals			11,250 (LC)	1520 (LC)	< 50	< 250	< 1000
	D2 Only applies to the VU category. Restricted area of occupancy or number of locations with a plausible future threat that could drive the taxon to CR or EX in a very short time.			276 km^2^ (LC); 78 km^2^ (LC)*; Nº loc. >10	32 km^2^ (LC); 9 km^2^ (VU)*; Nº loc. 8 (LC)			AOO < 20 km^2^/nº loc. ≤5
E Quantitative Analysis		-		No data available		≥ 50% in 10 years/3 gen.	≥ 20% in 20 years/5 gen.	≥ 10% in 100 years
Conservation status and codes		-		EN B2ab(iii)	EN B2ab(iii); C2a(ii) CR B2ab(iii)*			

*For both taxa, we have also calculated the area of occupancy (AOO) using 1 × 1 km (1 km [2]) cells, in addition to the IUCN standardised 2 × 2 km cells

However, we found new occurrences, such as small populations of *L. azoricus* in Santa Maria, and small restored populations in Pico. New locations were also found or rediscovered for *A. vidalii*. Since its occurrence area is much larger than that of *L. azoricus*, the total sampled areas for both species differ, but only due to the rarity of *L. azoricus*, not to sampling bias. As planned, we managed to sample plots with or without these two taxa, a clear example being the huge number of samples performed in Graciosa, where only one plot with *A. vidalii* was found. Nonetheless, it is possible that we have not found all the target taxa populations, since we aimed to sample and not to completely survey the coastline.

Missing areas would correspond mainly to vertical cliffs covered by native or invasive coastal scrubland, with large boulder beaches at sea level. Further sampling was out of our project capabilities, due to logistic, funding and time constraints, and could only be undertaken if regional entities would support boat access to coastal areas inaccessible by land.

It can be argued that the herbaceous vegetation at coastal areas is mainly dominated by generalist taxa, and that *L. azoricus* and *A. vidalli* are not truly coastal plants. We used a pragmatic approach that considered all herbaceous species present in the coast, above sea level, and below coastal scrubland (either natural or partially anthropogenic) [59] or anthromes - although this strip could be wider or narrower depending on site conditions (e.g., contour, human activities surrounding the area). However, our target species showed to be clearly restricted to this coastal vegetation belt, on highly exposed sea cliffs or rocky substrates, with incipient nutrients [60] and often together with *F. petraea* and *C. maritimum* [25, 26, 61], even when found at higher locations (100 m) such as at sea cliffs with gentler slopes (e.g., Corvo island).

### Recent changes in the coastal flora

*Festuca petraea* was the most frequent coastal taxon in our study, forming coastal meadows [59], but also found in dunes and chamaephyte rocky shore communities [24]. The present taxonomic circumscription differentiates it from *F. francoi* Fern.Prieto, C.Aguiar, E.Días & M.I.Gut, an endemic taxon found at higher elevations [32].

Overall, naturalised and invasive taxa, when considered together, were clearly the most frequent across all plot types, as previously reported [16]. This is linked to the occurrence of many generalist taxa adapted to natural or anthropogenic disturbance (e.g., *Portulaca oleracea*,* Sonchus* spp.), exhibiting subcosmopolitan distributions [16]. Although ocean-related disturbance leads to harsh coastal environments where non-indigenous taxa could have more difficulties to establish and thrive [62], our results show a tendency for an expansion of these taxa. This was also confirmed through the indicator species analysis, where alien taxa showed a relevant indicator value.

The occurrence of a high number of generalist, non-indigenous species on the coastal habitats, is leading to alterations in plant composition, cover, species richness, diversity, and evenness, due to disassembly processes [63], explaining the lack of support for the existence of well-defined coastal plant communities in the cluster analysis. While indigenous species still thrive in coastal plant communities of halophytic or lithophytic character [61], these are becoming scarce, given the expansion of non-indigenous taxa, with many invasive species (e.g., *A. donax*, *C. edulis*, *A. americana*, *P. undulatum*) [38] becoming dominant and invading large areas near the coast, where only a few endemic plant taxa survive, often at marginal habitats.

### Life forms and ecological adaptations

Many coastal endemic taxa (e.g., *Azorina vidalii*, *Euphorbia azorica*,* Limonium diasii*,* Spergularia azorica*,* Tolpis* spp.) were often observed [24], contributing to the considerable frequence of chamaephytes. The presence of endemic chamaephytes in island floras could be the result of secondary woodiness, prompting island taxa to longer life cycles and sturdy woody habits, in detriment of herbaceous habits [64]. However, hemicryptophytes and therophytes dominated the studied areas, due to the frequent presence of invasive, generalist taxa, as seen elsewhere [16].

As expected, our results showed that halophytes are still relevant in Azorean coastal plant communities [24, 61]. However, we also found many generalist taxa in control plots, but also in communities with *A. vidalii*. This resulted from their wider geographical and ecological niche, in their ability to thrive in different types of habitats, which could be exacerbated by climate change [65].

#### Azorina vidalii

We found that *A. vidalii* still commonly occurs in the Azorean coasts, in varied conditions, particularly at the base of sea cliffs [24], although not being restricted to the *Euphorbio azoricae-Festucion petraeae* alliance [27], supporting a broad ecology [24]. At sea level it is frequently found at *Festuca* meadows and other halophytic chamaephyte coastal communities from cliffs and rolled pebbled beaches [24, 26]. Less frequently, it was found at the margins of coastal scrubland or lowland juniper stands in Pico island [61], or more rarely, at higher elevation and inland areas, with reduced salinity and lower temperatures (e.g., Corvo and Faial islands) [24, 66]. Nonetheless, at *A. vidalii* plots we confirmed a high prevalence of non-indigenous taxa [16, 19] since it was often found at the margins of disturbed habitats (e.g., dense stands of *Arundo donax* or of other invasive species). Finally, we found some distinction between plots with *A. vidalli* and without both target plants, namely, a larger heterogeneity of species composition in the latter, as evident in the numerical ecology analyses (NMDS and Bray-Curtis).

#### Lotus azoricus

We confirmed the rarity of *L. azoricus* in the Azorean coasts, being restricted to highly exposed plant communities in difficult access areas (e.g., high elevation coastal cliffs in Santa Maria island), where it likely escapes the intense human disturbance [19, 21] found at flatter areas. It also occurs in volcanic substrates, highlighting the role of endemic taxa in the primary succession [67]. It was, in some cases, found in well-preserved halo-xerophytic communities (e.g., in Pico island), with *F. petreae* and *Plantago coronopus*, in salty-slime or clay deposits [61], with high levels of endemic species, reduced disturbance, and some biotic resistance towards invaders [68]. However, the largest population of this species, located in Ponta do Castelo, Santa Maria, was found among several invasive plants (see below). Plots with *L. azoricus* or with both target species were rare, the latter only occurring at Ponta do Castelo, in Santa Maria. This island presents relatively higher temperatures and less rainfall [69], favouring the occurrence of halo-xerophytic communities where *L. azoricus* and *A vidalii* appeared with *F. petreae* but also with *Carpobrotus edulis* and *Agave americana* [61].

### Environmental descriptors

Our results showed some level of nutrient enrichment of the soils, namely, high levels of extractable phosphorus in plots with both target endemics, at Santa Maria island (5 out of 6 plots). This suggests that agriculture, animal husbandry, and the presence of seabird colonies [61, 70] might be causing soil eutrophication. This, together with favourable climate, likely allows the expansion of eutrophication adapted taxa, potentially affecting island plant assemblages [67], and threatening the endemic halophytes present at the transition between marine and terrestrial ecosystems.

Climate appears as a relevant factor for the establishment and thrive of *L. azoricus*. According to the PCA and binary regression analyses, it was negatively associated with higher precipitation levels, justifying its common occurrence in warmer and drier habitats, contributing to its restricted and fragmented occurrence, mainly in Santa Maria Island [69]. This might be, however, related with its phenology, namely the onset of flowering [21].

In contrast, *A. vidalii* was found at places with wider climatic variation (i.e., thermal amplitude), from drier habitats, such as those of *L. azoricus*, to areas with higher precipitation and humidity levels [69]. But also, in a variety of substrates (from almost vertical cliffs, to rolled pebble beaches and soil filled rock crevices), appearing also on shallow soil deposits [61].

A large proportion of control plots were observed in sand substrates, probably linked with the human expansion in these areas, placing sandy shores among the most invaded terrestrial environments in Europe [71].

### Threats

Invasive species were the most frequent threat observed, with many known coastal invaders, such as *Carpobrotus edulis* and *Arundo donax* [38], accompanying indigenous taxa. While a worldwide concern [4, 7], their occurrence is intrinsically linked to human disturbance [72]. In the Azores, the proliferation of invasive species in coastal areas is related with traditional land use and, more recently, with the expansion of human infrastructures and economic activity [16, 73]. Abandonment of agricultural land allowed the expansion of deliberately introduced species in the coast, previously used as hedgerows (e.g., *Arundo donax*,* Metrosideros robusta* or *Tamarix africana*) [16, 38].

We found that another important threat is the expansion of pastureland to low elevation, further constraining habitat availability for coastal plants, often already reduced to a very narrow belt above sea level or restricted to coastal cliffs, as observed in Santa Maria Island, for both target species. Additional threats arise from free roaming animals that can graze or trample on the populations of rare endemic coastal plants, reducing native plant cover and opening space for non-indigenous opportunists [34]. *Lotus azoricus* is affected by cattle grazing, rabbits and rats [61], which negatively impact fitness and seed production [74].

We observed the occurrence of construction work near several populations of the target species, including threatened populations of *L. azoricus*. Increased economic activity, expansion of human activities and construction of infrastructures in coastal areas often result in habitat destruction and population fragmentation [4–6], raising new challenges to the survival of coastal endemic plants worldwide [3, 8]. The occurrence of illegal waste disposal in coastal cliffs, beaches or close to water streams, potentially affects marine species [75], degrades indigenous plants habitat [73], and facilitates the spread of invasive plant taxa. Conservative evolution on island plants often resulted in increased susceptibility to anthropic disturbance, and decreased defences against herbivory [76].

Several sea level populations of *A. vidalii* are threatened due to climate change and warming, which can raise sea level and intensify the occurrence of extreme weather events, leading to coastal flooding [1, 10] and to the potential loss of unique genetic characteristics of this species [29]. Previous work suggests that, due to climate change, the suitable climate space of *A. vidalii* could decline [77].

Direct sea exposure, through hydric stress, and anthropic disturbance can promote the erosion of plant fixing substrates [19, 21], triggering the occurrence of landslides and exacerbating natural erosion [78], thereby facilitating the establishment of naturalised taxa. Besides coastal disturbance, the mild Azorean climate might have also facilitated the proliferation of non-indigenous, generalist plants [69], which could still be aggravated by global warming [9, 77].

### Conservation status and prospects

Despite 24.1% of the Azorean territory is within protected areas, our results showed that less than half of the occurrences of *A. vidalii* were covered, while almost all *L. azoricus* populations in Santa Maria and São Jorge were covered, but not most of its populations in Pico. Despite the important conservation areas found in this island, nearly one third of natural habitat patches were found outside protected areas, under considerable degradation [11]. This is worrying, given the important presence of *L. azoricus* and *A. vidalii* in Pico.

Reassessing those areas for conservation will be vital for the preservation of both taxa in the larger islands, whose populations are more susceptible to threats, such as invasive species and others mentioned above, to avoid further losses due to anthropogenic disturbance [79]. Additionally, the frequent occurrence of both taxa within protected landscapes does not represent effective conservation, due to the low level of restrictions. Monitoring of these species inside and outside of protected areas is fundamental [79], since many impacting activities like agriculture or animal husbandry expanded almost to sea level. Outside protected areas, endemic plants are even more susceptible due to the lack of monitoring and impact assessments [79].

Previous evaluations considered *A. vidalii* as an Endangered species [22], while the conservation status of *L. azoricus* in Santa Maria island was Vulnerable [21]. Our evaluation of the conservation status of both species resulted in an Endangered (EN) status, showing a stable trend in the conservation status of these species. Given the rarity of *L. azoricus* [19], we argue that the standardised 2 × 2 km cells recommended by the IUCN [57] for determining the Area of Occupancy are likely not suitable to access its conservation status, since when using 1 × 1 km cells, we obtained Critically Endangered (CR) for *L. azoricus*.

The results provided by this research reinforce the need for active conservation measures for both species, but mostly for *L. azoricus*. The control of invasive taxa, training of municipality and environmental workers, and the restriction of cattle access should be undertaken [21, 80]. The involvement of local communities should also be a priority in monitoring and cleaning of trash disposals and trampling [75]. Citizen science initiatives that aim to instil local populations with conservation behaviours have generated positive outcomes elsewhere [81]. Finally, the role of botanic gardens in providing back-up materials for eventual *ex situ* conservation actions (e.g., Life Vidalia project) and the use of molecular genetic approaches to assess extinction risk and detect reduced genetic variation and inbreeding among populations will be particularly important for the conservation of these endemic species.

## Conclusions

Our work raises significant ecological questions regarding the current definition of the coastal herbaceous communities in the Azores. The communities previously described in the literature [23–27] are becoming rare, with indigenous plant taxa being restricted to a narrow vegetation belt constrained by sea level below and by coastal scrubland or anthromes above.

While endemic chamaephytes and halophytes still are an important component of these communities (e.g., in *Festuca* meadows, rocky chamaephyte communities and halo-xerophytic communities) [24, 61], we are observing an increased presence of generalist non-indigenous taxa, contributing for the homogenisation of the coastal plant communities. At several locations, the halophytic plant communities are at stake due to expansion of non-indigenous plants, despite the harsh coastal conditions.

Although environmental factors like dry climate, high salinity, poor nutrient availability and rocky substrates are still important ecological factors shaping Azorean coastal herbaceous plant communities [60, 69], increased anthropogenic disturbance and the expansion of highly competitive invasive taxa [16, 38] has gained importance. Increased anthropogenic disturbance derived from the expansion of economic activities [73] into the coastal areas is expected to continue in future years. Thus, natural coastal habitats are becoming scarce, due to the narrowing and replacement of the respective vegetation belt. This makes conservation and monitoring activities both inside and outside protected areas a priority, and suggests the need to periodically reevaluate the design of coastal protected areas.

The conservation status of these two species have not deteriorated, remaining as Endangered, suggesting that restoration initiatives were useful to avoid their further decline. Therefore, it is essential to continue to raise awareness for the conservation of the Azorean coastal plant communities [19].

### Electronic supplementary material

Below is the link to the electronic supplementary material.

Supplementary Material 1

## Data Availability

The datasets used and/or analysed during the current study are available from the corresponding author on reasonable request.
