# Supplementary material for: Anthropogenic disturbance has altered the habitat of two Azorean endemic coastal plants
Source: BMC Ecol Evol. 2024 Aug 20;24:111. doi: 10.1186/s12862-024-02300-8 (PMC11337623; doi:10.1186/s12862-024-02300-8)
Supplement: Supplementary file 1 — Supplementary Material 1 [file 12862_2024_2300_MOESM1_ESM.docx]

Supplementary Figure S1. Percentage values of plant cover for species (> 1%) found in 231 plots made in coastal habitats in nine islands of the Azores.

Supplementary Figure S2. Frequency. in percentage. of species (starting in *L. azoricus*) found in 231 plots made in coastal habitats in nine islands of the Azores.


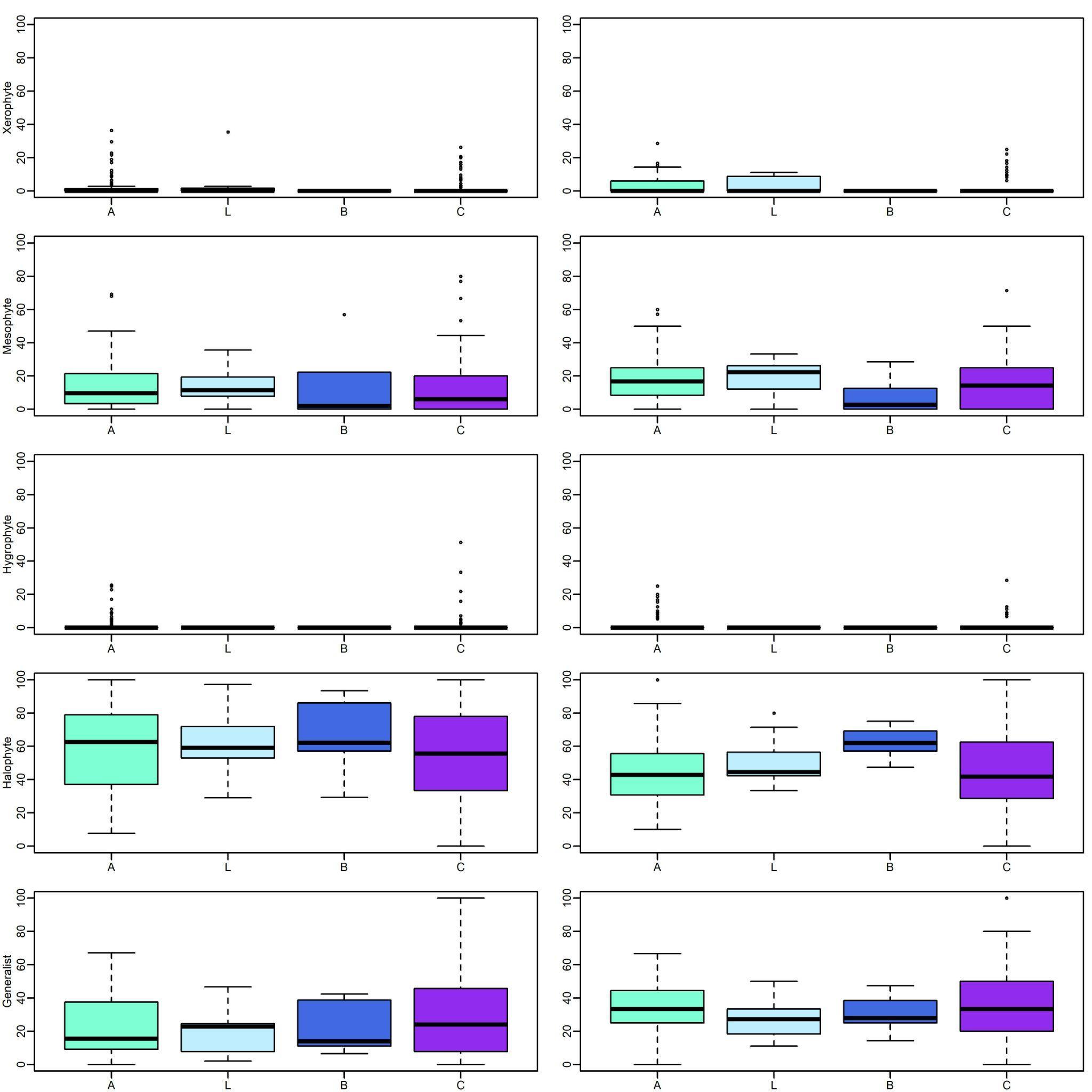


Supplementary Figure S3. Boxplots of ecology types across plot types, based on 231 plots sampled in coastal areas in the nine Azores islands. Plot types: A – including *Azorina vidalii*, L – including *Lotus azoricus*, B – including both species, C – controls without both species.


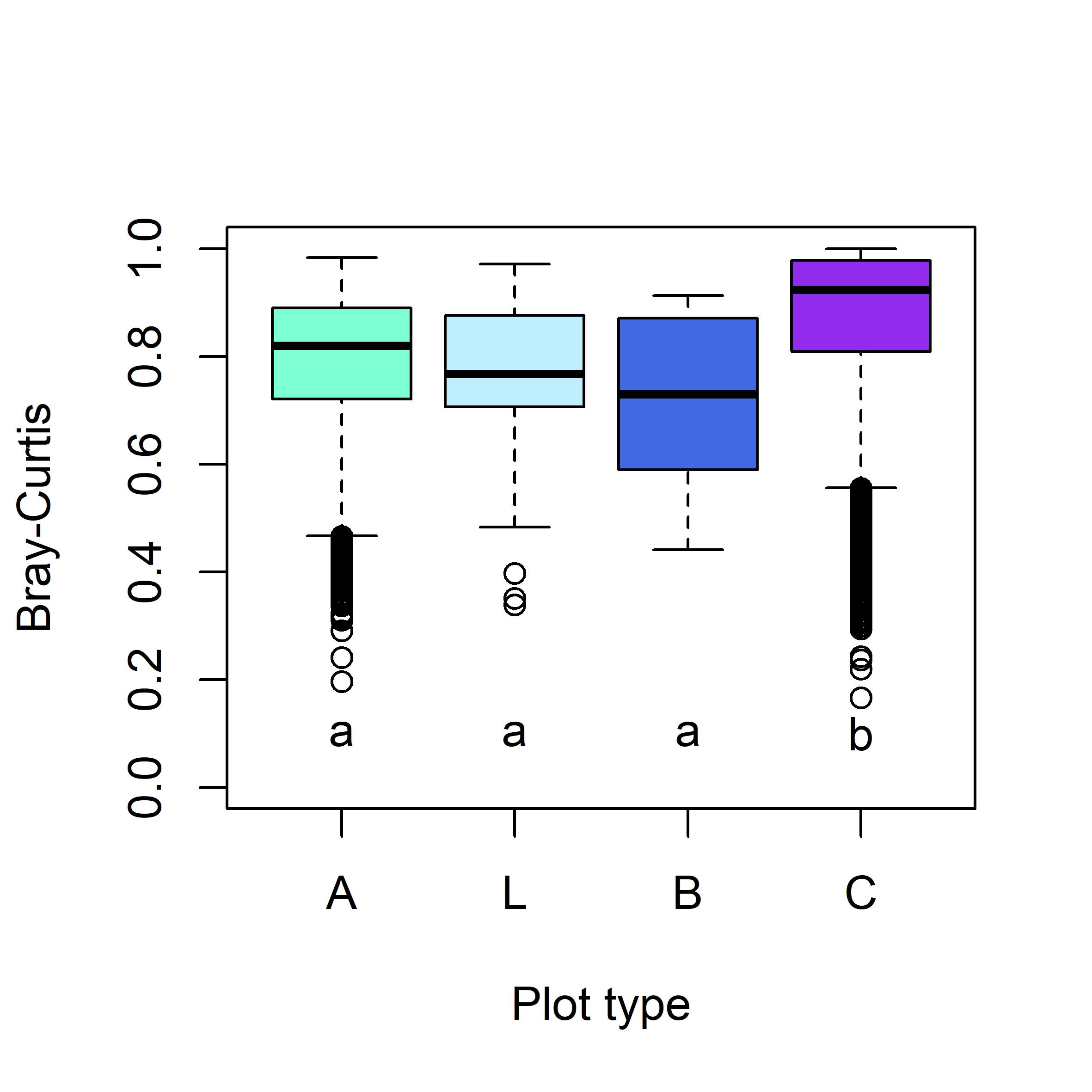


Supplementary Figure S4. Bray-Curtis dissimilarity index calculated separately by plot type, based on 231 plots sampled in coastal areas in the nine Azores islands. Plot types: A – including *Azorina vidalii*, L – including *Lotus azoricus*, B – including both species, C – controls without both species. Different letters indicate significant differences (p<0.05); Results of the multiple comparison test applied after KW-test.


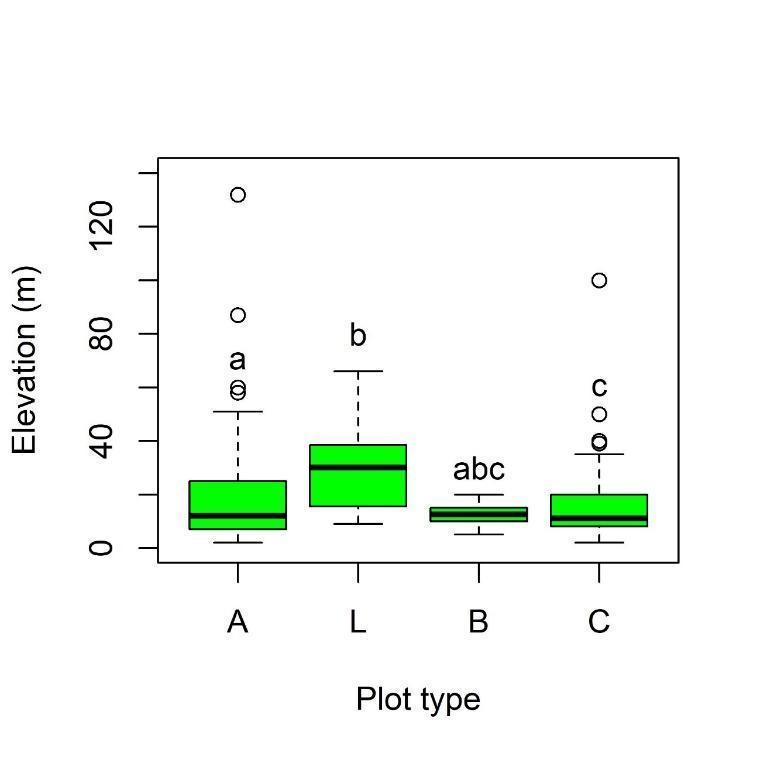


Supplementary Figure S5. Boxplot of elevation across plot types, based on 231 plots sampled in coastal areas in the nine Azores islands. Plot types: A – including *Azorina vidalii*, L – including *Lotus azoricus*, B – including both species, C – controls without both species. Different letters indicate significant differences (p<0.05); Results of a multiple comparison test applied after the Kruskal-Wallis test.


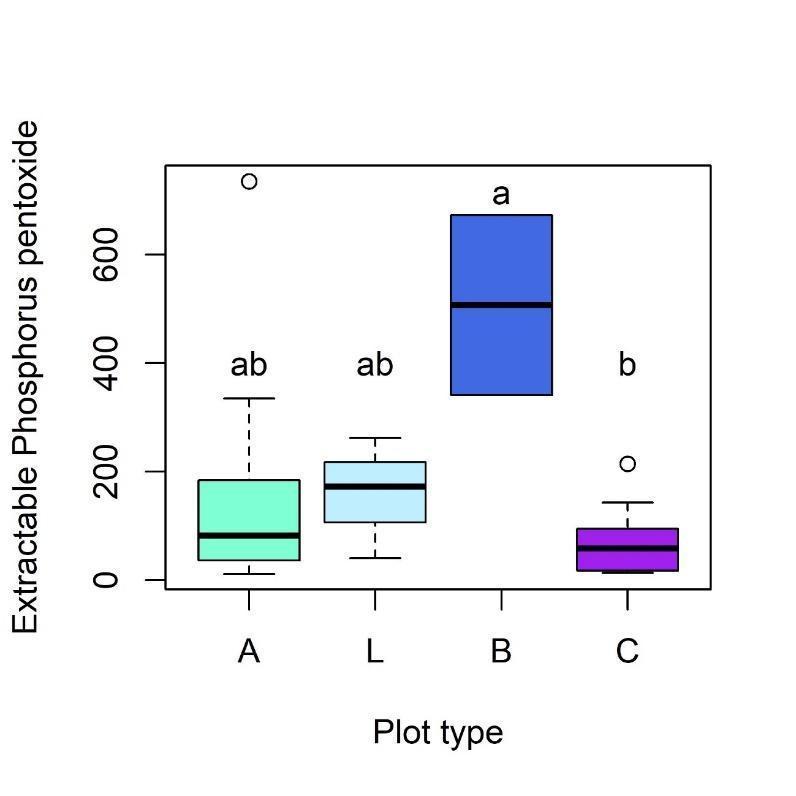


Supplementary Figure S6. Boxplot of the Extractible content of P_2_O_5_ by Eletrolite Replacement (kg^-1^), based on 231 plots sampled in coastal areas in the nine Azores islands. Plot types: A – including *Azorina vidalii*, L – including *Lotus azoricus*, B – including both species, C – controls without both species. Different letters indicate significant differences (p<0.05); Results of a multiple comparison test applied after the Kruskal-Wallis test.


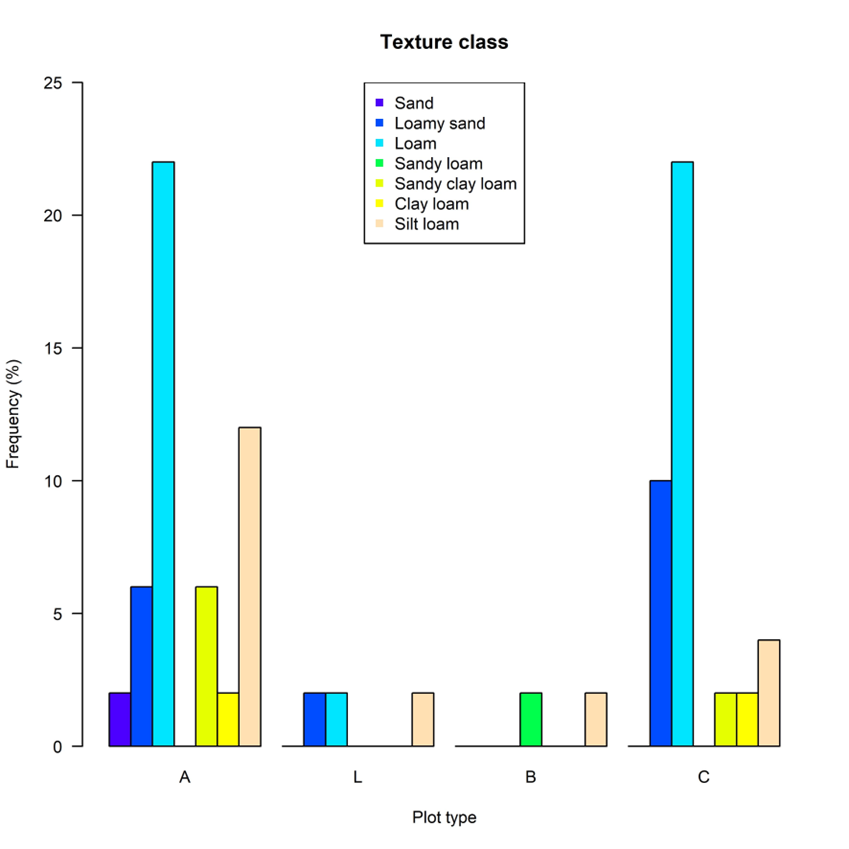
Supplementary Figure S7. Diagram of the frequency percentages of different texture classes among plot types, based on 231 plots sampled in coastal areas in the nine Azores islands. Plot types: A – including *Azorina vidalii*, L – including *Lotus azoricus*, B – including both species, C – controls without both species.


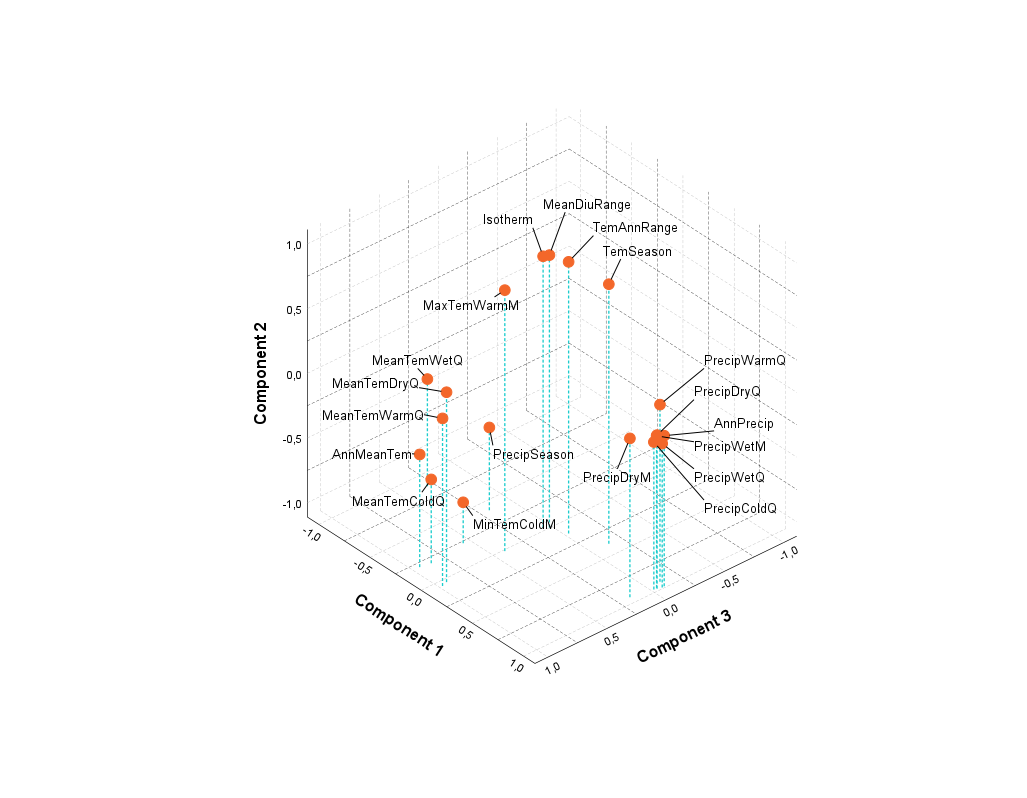


Supplementary Figure S8. Principal Component Analysis applied to the 19 bioclimatic variables, resulting in three components explaining 48, 26, and 20% of the variance, respectively. Abbreviations meanings are in Supplementary Table S2.


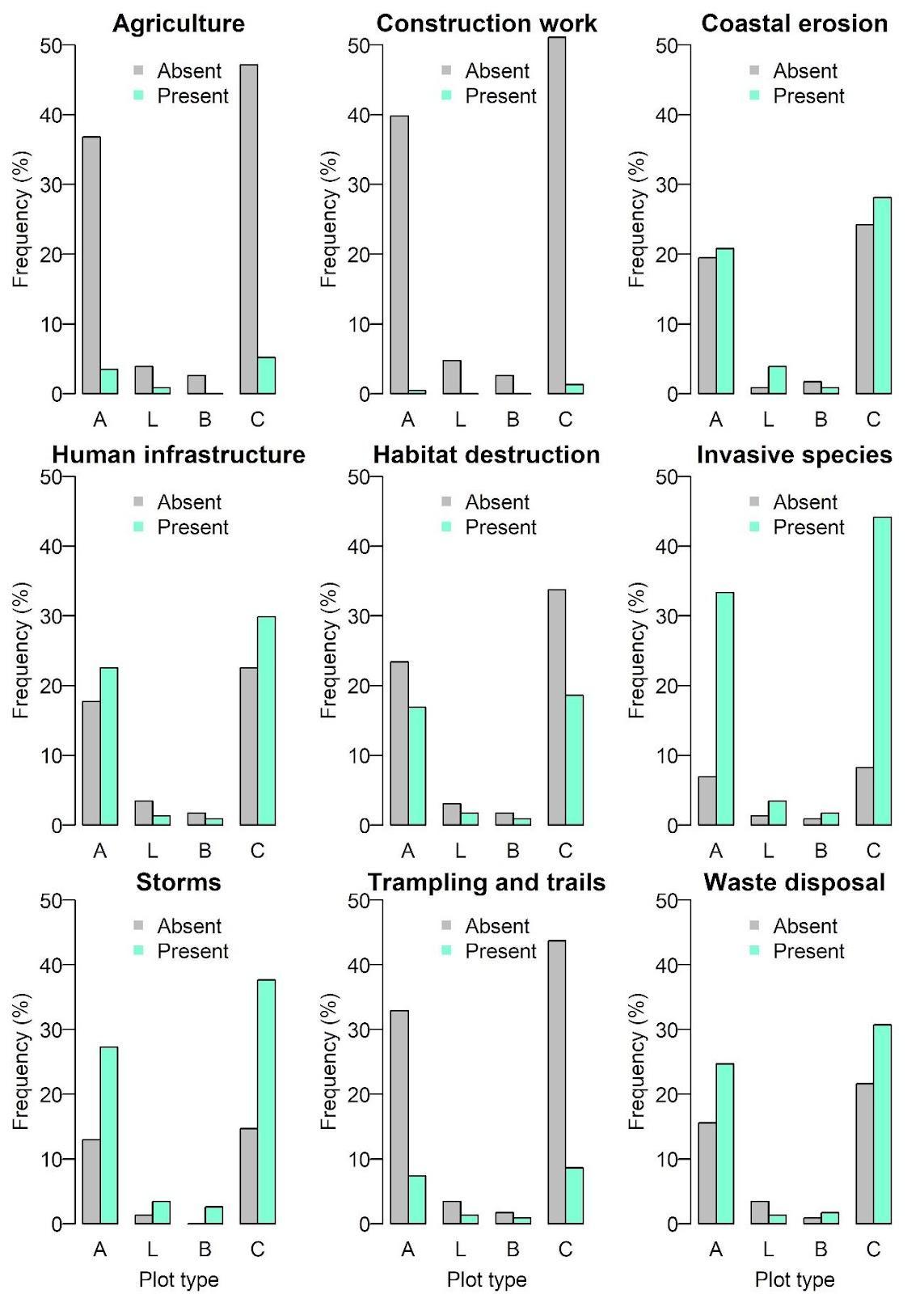


Supplementary Figure S9. Frequency of several threats found in 231 plots made in coastal areas on nine islands of the Azores. Plot types: A –*Azorina vidalii*, L – *Lotus azoricus*, B –both species, C – control.

Supplementary Table S1. List of taxa collected, with colonisation status, life forms, voucher IDs, occurrence islands and frequency.

| Species | Colonisation status | Life forms | Habitat ecology | Voucher ID | Islands of occurrence | Frequency(%) |
| --- | --- | --- | --- | --- | --- | --- |
| *Acacia melanoxylon* R.Br. | invasive | phanerophyte | mesophyte |  | SMA, SMG, TER, GRA, SJO, PIC, FAI, FLO | 0.4329 |
| *Adiantum capillus-veneris* L. | native | hemicryptophyte | mesophyte |  | SMA, SMG, TER, GRA, SJO, PIC, FAI, FLO | 1.298701 |
| *Agave americana* L. | invasive | phanerophyte | xerophyte |  | SMA, SMG, TER, GRA, PIC, FAI, FLO | 3.463203 |
| *Agave attenuata* Salm-Dyck | naturalised | phanerophyte | xerophyte |  | TER, SJO | 0.4329 |
| *Ageratina adenophora* (Spreng.) R.M.King & H.Rob. | invasive | hemicryptophyte | generalist |  | SMG, TER, SJO, PIC, FAI | 0.865801 |
| *Ailanthus altissima* (Mill.) Swingle | invasive | phanerophyte | mesophyte |  | SMA, SMG, TER, GRA, SJO, PIC, FAI, FLO | 0.4329 |
| *Aloe arborescens* Mill. | naturalised | phanerophyte | xerophyte |  | SMG, TER, GRA, SJO, FAI | 1.298701 |
| *Amaranthus blitum* L. | invasive | therophyte | mesophyte |  | All islands | 0.4329 |
| *Ammi majus* L. | naturalised | therophyte | mesophyte | 4317; 4362 | SMA, SMG, TER, GRA, SJO, PIC, FAI, FLO | 0.4329 |
| *Ammi seubertianum* (H.C.Watson) Trel. | endemic | hemicryptophyte | halophyte |  | SMA, SMG, SJO, FAI, COR | 1.298701 |
| *Anredera cordifolia* (Ten.) Steenis | naturalised | phanerophyte | generalist |  | SMA, SMG, TER, GRA, SJO, PIC, FAI, FLO | 2.164502 |
| *Apium graveolens* L. | naturalised | hemicryptophyte | hygrophyte |  | All islands | 5.627706 |
| *Araujia sericifera* Brot. | invasive | phanerophyte | generalist |  | SMA, SMG, TER, GRA, SJO, PIC, FAI, FLO | 1.298701 |
| *Arundo donax* L. | invasive | phanerophyte | generalist |  | All islands | 12.98701 |
| *Asplenium marinum* L. | native | hemicryptophyte | halophyte |  | All islands | 6.493506 |
| *Atriplex prostrata* Boucher ex DC. | native | therophyte | generalist |  | All islands | 27.70563 |
| *Austrocylindropuntia subulata* (Engelm.) Backeb. | naturalised | chamaephyte | xerophyte |  | TER | 0.4329 |
| *Azorina vidalii* (H.C.Watson) Feer | endemic | chamaephyte | halophyte | 4330-4336; 4343-4360; 4364-4374 | All islands | 42.85714 |
| *Beta maritima* L. | native | therophyte | halophyte |  | SMA, SMG, TER, GRA, SJO, PIC, FAI | 2.597403 |
| *Brachypodium sylvaticum* P.Beauv. | native | hemicryptophyte | generalist |  | All islands | 6.926407 |
| *Briza minor* L. | naturalised | therophyte | generalist |  | All islands | 0.4329 |
| *Bromus hordeaceus* subsp. *divaricatus* (Bonnier & de Layens) Kerguélen | naturalised | therophyte | generalist |  | SMA, SMG, TER, GRA, SJO, PIC, FAI | 0.865801 |
| *Calendula suffruticosa* Vahl | naturalised | chamaephyte | generalist |  | SMA | 1.298701 |
| *Calluna vulgaris* (L.) Hull | native | phanerophyte | mesophyte |  | All islands | 0.4329 |
| *Calystegia sepium* R.Br. subsp. *sepium* | naturalised | hemicryptophyte | generalist |  | SMA, SMG, TER, SJO, PIC, FAI, FLO, COR | 1.298701 |
| *Carduus tenuiflorus* W.M.Curtis | naturalised | chamaephyte | generalist |  | All islands | 0.4329 |
| *Carex hochstetteriana* J.Gay ex Seub. | endemic | hemicryptophyte | mesophyte |  | SMA, SMG, TER, SJO, PIC, FAI, FLO, COR | 0.4329 |
| *Carex vulcani* Hochst. ex Seub. | endemic | hemicryptophyte | mesophyte |  | SMG, TER, SJO, PIC, FAI, FLO | 0.865801 |
| *Carpobrotus edulis* (L.) N.E.Br. | invasive | chamaephyte | halophyte |  | All islands | 16.45022 |
| *Chenopodiastrum murale* (L.) S.Fuentes, Uotila & Borsch | invasive | therophyte | mesophyte |  | All islands | 1.731602 |
| *Chenopodium album* L. | invasive | therophyte | mesophyte |  | All islands | 3.030303 |
| *Cichorium intybus* L. | naturalised | hemicryptophyte | mesophyte |  | SMA, SMG, TER, GRA, SJO, FAI, FLO, COR | 0.4329 |
| *Coleostephus myconis* (L.) Rchb.f. | naturalised | therophyte | mesophyte |  | SMA, SMG, TER, GRA, SJO, PIC, FAI, FLO | 0.865801 |
| *Colocasia esculenta* (L.) Schott | invasive | geophyte | hygrophyte |  | All islands | 0.4329 |
| *Convolvulus arvensis* L. | naturalised | hemicryptophyte | mesophyte |  | SMA, TER, GRA, SJO, COR | 0.4329 |
| *Corema azoricum* (P.Silva) Rivas Mart., Lousã, Fern.Prieto, E.Dias, J.C.Costa & C.Aguiar | endemic | phanerophyte | halophyte | 4342 | SMG, GRA, SJO, PIC, FAI | 0.4329 |
| *Crithmum maritimum* L. | native | hemicryptophyte | halophyte |  | All islands | 34.63203 |
| *Crocosmia* x *crocosmiiflora* (Nicholson) N.E.Br. | invasive | geophyte | generalist |  | SMG | 1.298701 |
| *Cymbalaria muralis* G.Gaertn., B.Mey. & Scherb. | naturalised | chamaephyte | mesophyte |  | SMA, SMG, TER, SJO, PIC, FAI, FLO, COR | 0.865801 |
| *Cynodon dactylon* (L.) Pers. | invasive | hemicryptophyte | generalist |  | All islands | 10.38961 |
| *Cyperus eragrostis* Lam. | naturalised | hemicryptophyte | mesophyte |  | All islands | 1.731602 |
| *Cyperus esculentus* L. | invasive | chamaephyte | generalist |  | All islands | 4.329004 |
| *Cyperus rotundus* L. | invasive | chamaephyte | generalist |  | All islands | 2.164502 |
| *Cyrtomium falcatum* (L.f.) C.Presl | invasive | chamaephyte | mesophyte |  | All islands | 33.76623 |
| *Dactylis glomerata* L. | naturalised | hemicryptophyte | mesophyte |  | All islands | 1.731602 |
| *Datura stramonium* L. | naturalised | therophyte | mesophyte |  | All islands | 0.4329 |
| *Daucus carota* L. | native | hemicryptophyte | generalist |  | All islands | 21.21212 |
| *Delairea odorata* Lem. | naturalised | phanerophyte | mesophyte |  | SMA, SMG, TER, GRA, SJO, PIC, FAI, FLO | 0.865801 |
| *Digitaria ciliaris* (Retz.) Koeler | naturalised | therophyte | generalist |  | All islands | 3.896104 |
| *Digitaria sanguinalis* (L.) Scop. | invasive | therophyte | generalist |  | All islands | 3.463203 |
| *Drosanthemum floribundum* (Haw.) Schwantes | invasive | chamaephyte | halophyte |  | All islands | 5.194805 |
| *Dysphania ambrosioides* (L.) Mosyakin & Clemants | naturalised | therophyte | mesophyte |  | All islands | 1.731602 |
| *Ecballium elaterium* A.Rich. | naturalised | hemicryptophyte | generalist |  | SMA, SMG, TER, GRA, SJO, PIC | 0.4329 |
| *Echium plantagineum* L. | naturalised | chamaephyte | mesophyte |  | SMA, SMG, TER, GRA, SJO, PIC, FAI, FLO | 1.298701 |
| *Eleusine indica* (L.) P.Gaertn. subsp. *indica* | invasive | therophyte | mesophyte |  | All islands | 1.298701 |
| *Equisetum telmateia* Ehrh. | native | geophyte | hygrophyte |  | SMA, SMG, TER, SJO, PIC, FAI, FLO | 0.4329 |
| *Erica azorica* Hochst. ex Seub. | endemic | phanerophyte | generalist |  | All islands | 10.38961 |
| *Erigeron bonariensis* L. | invasive | therophyte | generalist |  | All islands | 10.82251 |
| *Euphorbia azorica* Hochst. ex Seub. | endemic | chamaephyte | halophyte |  | All islands | 31.16883 |
| *Euphorbia maculata* L. | naturalised | therophyte | generalist |  | All islands | 5.627706 |
| *Farfugium japonicum* (L.) Kitam. | cultivada | phanerophyte | generalist |  | SMA, PIC | 0.4329 |
| *Festuca petraea* Guthn. ex Seub. | endemic | hemicryptophyte | halophyte |  | All islands | 51.51515 |
| *Ficus carica* L. | naturalised | phanerophyte | mesophyte |  | All islands | 0.865801 |
| *Foeniculum vulgare* Mill. | naturalised | hemicryptophyte | mesophyte |  | All islands | 1.731602 |
| *Frankenia laevis* L. | native | phanerophyte | halophyte |  | SJO, COR | 1.731602 |
| *Frankenia pulverulenta* L. | native | therophyte | halophyte |  | All islands | 3.030303 |
| *Fumaria muralis* W.D.J.Koch subsp. *muralis* | naturalised | therophyte | generalist |  | SMA, SMG, TER, GRA, SJO, PIC, FAI, FLO | 3.463203 |
| *Gastridium ventricosum* (Gouan) Schinz & Thell. | naturalised | therophyte | halophyte |  | SMA, SMG, TER, GRA, SJO, PIC, FAI, FLO | 17.74892 |
| *Gaudinia coarctata* (Link) T.Durand & Schinz | endemic | therophyte | halophyte |  | All islands | 12.12121 |
| *Gaudinia fragilis* (L.) P.Beauv. | naturalised | therophyte | halophyte |  | SMA, SMG, TER, GRA, SJO, PIC, FAI | 1.298701 |
| *Gazania rigens* (L.) Gaertn. | naturalised | chamaephyte | halophyte |  | SMG, TER, GRA, PIC, FAI, FLO | 0.4329 |
| *Glebionis segetum* Fourr. | naturalised | therophyte | generalist |  | All islands | 0.865801 |
| *Hedera azorica* Carrière | endemic | phanerophyte | mesophyte |  | All islands | 2.597403 |
| *Helianthus tuberosus* L. | naturalised | geophyte | mesophyte |  | SMA, SMG, GRA, FAI, FLO | 0.4329 |
| *Helichrysum luteoalbum* (L.) Rchb. | native | therophyte | generalist |  | All islands | 8.658009 |
| *Heliotropium curassavicum* L. | naturalised | chamaephyte | halophyte |  | SMG | 0.4329 |
| *Helminthotheca echioides* (L.) Holub | naturalised | chamaephyte | generalist |  | All islands | 4.761905 |
| *Holcus lanatus* L. | invasive | hemicryptophyte | mesophyte |  | All islands | 10.82251 |
| *Holcus rigidus* Hochst. ex Seub. | endemic | hemicryptophyte | mesophyte |  | All islands | 0.865801 |
| *Hordeum murinum* subsp. leporinum (Link) Arcang. | naturalised | therophyte | generalist |  | All islands | 1.298701 |
| *Hypericum humifusum* L. | native | chamaephyte | generalist |  | All islands | 0.4329 |
| *Hypochaeris radicata* L. | invasive | hemicryptophyte | generalist |  | All islands | 0.4329 |
| *Ipomoea batatas* (L.) Lam. | naturalised | geophyte | mesophyte |  | SMA, SMG, SJO, FAI, FLO | 0.4329 |
| *Ipomoea indica* Merr. | invasive | geophyte | mesophyte |  | SMA, SMG, TER, GRA, SJO, PIC, FAI, FLO | 1.731602 |
| *Juncus acutus* L. | native | chamaephyte | halophyte |  | All islands | 11.68831 |
| *Juniperus brevifolia* (Seub.) Antoine | endemic | phanerophyte | mesophyte |  | SMG, TER, SJO, PIC, FAI, FLO, COR | 1.731602 |
| *Kickxia spuria* Dumort. subsp*. spuria* | naturalised | therophyte | mesophyte |  | All islands | 0.4329 |
| *Lantana camara* L. | invasive | phanerophyte | generalist |  | SMA, SMG, TER, GRA, SJO, PIC, FAI, FLO | 1.298701 |
| *Lathyrus aphaca* L. | naturalised | therophyte | generalist |  | SMA, SMG, PIC, FAI, FLO | 0.4329 |
| *Lathyrus tingitanus* L. | naturalised | therophyte | generalist |  | SMA, SMG, TER, GRA, PIC, FAI, FLO | 0.4329 |
| *Leontodon longirostris* (Finch & P.D.Sell) Talavera | native | chamaephyte | generalist |  | All islands | 8.658009 |
| *Lepidium coronopus* (L.) Al-Shehbaz | naturalised | therophyte | generalist |  | SMA, SMG, TER, GRA | 0.4329 |
| *Lepidium didymum* L. | naturalised | therophyte | generalist |  | All islands | 1.298701 |
| *Lepidium virginicum* L. | naturalised | therophyte | generalist |  | SMA, SMG, TER, GRA, SJO, PIC, FAI, FLO | 0.4329 |
| *Limonium diasii* Fern.Prieto, T.E.Díaz & C. Aguiar | endemic | chamaephyte | halophyte | 4311-4316; 4318-4328 | SMA, SMG, TER, PIC | 9.95671 |
| *Lobularia maritima*  (L.) Desv. | naturalised | chamaephyte | halophyte |  | All islands | 0.865801 |
| *Lolium multiflorum* Lam. | naturalised | chamaephyte | mesophyte |  | All islands | 6.926407 |
| *Lolium perenne* L. | naturalised | hemicryptophyte | mesophyte |  | All islands | 3.463203 |
| *Lolium rigidum* Gaudin | naturalised | therophyte | mesophyte |  | SMA, SMG, SJO, FAI, FLO, COR | 4.761905 |
| *Lotus azoricus* P.W.Ball | endemic | hemicryptophyte | halophyte | 4329; 4361 | SMA, SMG(?), SJO, PIC, FLO(?) | 7.359307 |
| *Lotus corniculatus* L. | naturalised | hemicryptophyte | generalist |  | All islands | 8.225108 |
| *Lotus creticus* L. | native | hemicryptophyte | generalist |  | TER | 3.896104 |
| *Lotus parviflorus* Desf. | naturalised | therophyte | generalist |  | SMA, SMG, TER, GRA, PIC, FAI, FLO, COR | 0.865801 |
| *Lotus pedunculatus* Cav. | naturalised | hemicryptophyte | generalist |  | All islands | 3.030303 |
| *Lotus subbiflorus* Lag. | doubtful | therophyte | generalist |  | All islands | 16.88312 |
| *Lysimachia arvensis* (L.) U.Manns & Anderb. subsp. arvensis | naturalised | therophyte | generalist |  | All islands | 10.38961 |
| *Malva multiflora* (Cav.) Soldano, Banfi & Galasso | naturalised | therophyte | generalist |  | All islands | 3.463203 |
| *Medicago lupulina* L. | naturalised | chamaephyte | generalist |  | All islands | 7.359307 |
| *Medicago polymorpha* L. | naturalised | therophyte | generalist |  | SMA, SMG, TER, GRA, SJO, PIC, FAI | 0.865801 |
| *Melilotus indicus* (L.) All. | naturalised | therophyte | generalist |  | SMA, SMG, TER, GRA, SJO, PIC, FAI, COR | 6.493506 |
| *Mentha aquatica* L. | native | hemicryptophyte | hygrophyte |  | SMA, SMG, TER, SJO, PIC, FAI, FLO, COR | 0.4329 |
| *Mentha suaveolens* Ehrh. | naturalised | hemicryptophyte | generalist |  | All islands | 0.4329 |
| *Mercurialis annua* L. | naturalised | therophyte | generalist |  | All islands | 0.4329 |
| *Mesembryanthemum cordifolium* L.f. | invasive | chamaephyte | xerophyte |  | All islands | 4.761905 |
| *Metrosideros excelsa* Gaertn. | invasive | phanerophyte | mesophyte |  | SMA, SMG, TER, GRA, PIC, FAI, FLO | 3.896104 |
| *Mirabilis jalapa* L. | naturalised | chamaephyte | generalist |  | All islands | 1.298701 |
| *Morella faya* (Aiton) Wilbur | native | phanerophyte | mesophyte |  | All islands | 3.463203 |
| *Myosotis maritima* Hochst. ex Seub. | endemic | hemicryptophyte | halophyte |  | SMA, TER, GRA, SJO, PIC, FAI, FLO, COR | 0.4329 |
| *Nasturtium officinale* W.T.Aiton | naturalised | geophyte | hygrophyte |  | All islands | 0.4329 |
| *Nothoscordum gracile* (Aiton) Stearn | naturalised | geophyte | mesophyte |  | All islands | 0.865801 |
| *Oenothera biennis* L. | naturalised | phanerophyte | mesophyte |  | SMG, TER, SJO, PIC, FAI, COR | 0.4329 |
| *Oenothera glazioviana* Micheli | naturalised | hemicryptophyte | mesophyte |  | SMA, SMG, TER, GRA, SJO, PIC, FAI, FLO | 0.865801 |
| *Opuntia stricta* (L.) Mill. | invasive | chamaephyte | xerophyte |  | SMG, FAI, FLO | 2.164502 |
| *Ornithopus pinnatus* (Mill.) Druce | native | therophyte | mesophyte |  | All islands | 3.463203 |
| *Osteospermum ecklonis* (DC.) Norl. | invasive | chamaephyte | mesophyte |  | SMG, TER, GRA, FAI | 3.030303 |
| *Oxalis corniculata* L. | naturalised | chamaephyte | generalist |  | All islands | 0.865801 |
| *Oxalis pes-caprae* L. | naturalised | geophyte | mesophyte |  | SMA, SMG, TER, GRA, SJO, PIC, FAI, FLO | 0.4329 |
| *Oxalis purpurea* L. | naturalised | geophyte | generalist |  | SMA, SMG, TER, GRA, SJO, PIC, FAI, FLO | 0.4329 |
| *Papaver rhoeas* L. | naturalised | therophyte | mesophyte |  | All islands | 0.4329 |
| *Papaver somniferum* L. *subsp. somniferum* | naturalised | therophyte | mesophyte |  | SMA, TER, GRA, SJO, PIC, FAI, FLO, COR | 0.4329 |
| *Parietaria judaica* L. | naturalised | chamaephyte | generalist |  | All islands | 13.41991 |
| *Paspalum dilatatum* Poir. | invasive | hemicryptophyte | mesophyte |  | All islands | 14.28571 |
| *Paspalum distichum* L. | invasive | hemicryptophyte | mesophyte |  | All islands | 0.865801 |
| *Paspalum urvillei* Steud. | invasive | hemicryptophyte | generalist |  | SMA, SMG, TER | 0.4329 |
| *Pericallis malvifolia* (L’Hér.) B.Nord. | endemic | hemicryptophyte | mesophyte |  | SMA, SMG, TER, SJO, PIC, FAI | 3.463203 |
| *Persicaria capitata* (Buch.-Ham. Ex D.Don) H.Gross | invasive | hemicryptophyte | hygrophyte |  | SMA, SMG, TER, SJO, PIC, FAI, FLO | 1.298701 |
| *Petroselinum crispum* (Mill.) Fuss | naturalised | hemicryptophyte | mesophyte |  | All islands | 0.865801 |
| *Phormium tenax* J.R.Forst. & G.Forst. | invasive | hemicryptophyte | xerophyte |  | All islands | 2.164502 |
| *Phytolacca americana* L. | invasive | hemicryptophyte | generalist |  | All islands | 6.060606 |
| *Picconia azorica* (Tutin) Knobl. | endemic | phanerophyte | mesophyte |  | SMA, SMG, TER, SJO, PIC, FAI, FLO, COR | 2.164502 |
| *Pittosporum tobira* W.T.Aiton | naturalised | phanerophyte | generalist |  | SMG, TER, GRA, SJO, FAI, FLO, COR | 2.164502 |
| *Pittosporum undulatum* Vent. | invasive | phanerophyte | generalist |  | All islands | 1.731602 |
| *Plantago coronopus* L. | native | hemicryptophyte | halophyte |  | All islands | 50.21645 |
| *Plantago lanceolata* L. | naturalised | hemicryptophyte | mesophyte |  | All islands | 8.225108 |
| *Plantago major* L. | naturalised | hemicryptophyte | mesophyte |  | All islands | 1.298701 |
| *Poa annua* L. | naturalised | chamaephyte | mesophyte |  | All islands | 0.865801 |
| *Polycarpon tetraphyllum* L. | naturalised | therophyte | generalist |  | All islands | 0.865801 |
| *Polypogon maritimus* Willd. | native | therophyte | mesophyte |  | All islands | 0.865801 |
| *Portulaca oleracea* L. | invasive | therophyte | generalist |  | All islands | 17.74892 |
| *Pteridium aquilinum* (L.) Kuhn | native | geophyte | generalist |  | All islands | 3.896104 |
| *Raphanus raphanistrum* L. | naturalised | therophyte | mesophyte |  | All islands | 0.4329 |
| *Reichardia picroides* Roth | naturalised | therophyte | halophyte |  | SMA | 6.493506 |
| *Rhaphiolepis indica* var*. umbellata* (Thunb. ex Murray) H.Ohashi | invasive | phanerophyte | generalist |  | SMG, TER, GRA, FLO | 1.298701 |
| *Ricinus communis* L. | invasive | phanerophyte | generalist |  | All islands | 1.298701 |
| *Rubus ulmifolius* Schott | invasive | phanerophyte | generalist |  | All islands | 1.731602 |
| *Rumex azoricus* Rech.f. | endemic | hemicryptophyte | hygrophyte |  | SMG, TER, SJO, FAI, COR | 0.4329 |
| *Rumex pulcher* L. subsp. *pulcher* | naturalised | hemicryptophyte | xerophyte |  | All islands | 1.298701 |
| *Sagina maritima* G.Don | native | therophyte | halophyte |  | All islands | 0.4329 |
| *Salpichroa origanifolia* (Lam.) Baill. | invasive | chamaephyte | hygrophyte |  | SMA, SMG, TER, GRA, SJO, PIC, FAI, FLO | 5.194805 |
| *Samolus valerandi* L. | native | hemicryptophyte | hygrophyte |  | SJO, FLO | 0.4329 |
| *Scrophularia auriculata* L. | naturalised | hemicryptophyte | hygrophyte |  | SMA, SMG, TER, SJO, FAI, FLO | 0.4329 |
| *Selaginella kraussiana* (Kunze) A.Braun | native | hemicryptophyte | hygrophyte |  | All islands | 1.298701 |
| *Setaria helvola* Roem. & Schult. | naturalised | therophyte | xerophyte |  | All islands | 1.731602 |
| *Setaria verticillata* (L.) P.Beauv. | naturalised | therophyte | xerophyte |  | All islands | 3.463203 |
| *Silene gallica* L. | naturalised | therophyte | generalist |  | All islands | 0.865801 |
| *Silene uniflora* Roth subsp*. uniflora* | native | hemicryptophyte | halophyte |  | SMG, TER, SJO, PIC, FAI, FLO, COR | 12.55411 |
| *Solanum mauritianum* Scop. | invasive | phanerophyte | generalist |  | SMA, SMG, TER, SJO, PIC, FAI, FLO | 0.865801 |
| *Solanum nigrum* L. | naturalised | therophyte | generalist |  | All islands | 1.298701 |
| *Solanum pseudocapsicum* L. | naturalised | phanerophyte | generalist |  | All islands | 0.4329 |
| *Solidago azorica* Hochst. ex Seub. | endemic | hemicryptophyte | mesophyte |  | All islands | 12.55411 |
| *Sonchus asper* Hill subsp. *asper* | naturalised | therophyte | generalist |  | All islands | 1.298701 |
| *Sonchus oleraceus* L. | naturalised | therophyte | generalist |  | All islands | 0.4329 |
| *Sonchus tenerrimus* L. | naturalised | therophyte | generalist |  | All islands | 46.75325 |
| *Sorghum halepense* Pers. | naturalised | hemicryptophyte | generalist |  | SMA, SMG, TER, GRA, FAI, FLO | 0.4329 |
| *Spergularia azorica* Lebel | endemic | chamaephyte | halophyte |  | All islands | 6.060606 |
| *Spergularia marina* (L.) Besser | naturalised | therophyte | halophyte | 4337 | All islands | 2.164502 |
| *Sporobolus africanus* (Poir.) Robyns & Tournay | naturalised | chamaephyte | xerophyte |  | All islands | 2.164502 |
| *Sporobolus pumilus* (Roth) P.M.Peterson & Saarela | invasive | chamaephyte | halophyte |  | SMA, SMG, TER, GRA, SJO, PIC, FAI, FLO | 6.060606 |
| *Stachys arvensis* L. | naturalised | therophyte | generalist |  | All islands | 0.4329 |
| *Stenotaphrum secundatum* (Walter) Kuntze | invasive | hemicryptophyte | halophyte |  | All islands | 3.896104 |
| *Symphyotrichum subulatum* var. *squamatum* (Spreng.) S.D.Sundb. | invasive | therophyte | halophyte |  | SMA, SMG, TER, GRA, SJO, PIC, FAI, FLO | 5.627706 |
| *Tamarix africana* Poir. | naturalised | phanerophyte | halophyte |  | All islands | 11.68831 |
| *Taraxacum officinale* F.H.Wigg. | naturalised | hemicryptophyte | generalist |  | TER, FAI | 0.4329 |
| *Tetragonia tetragonoides* (Pall.) Kuntze | invasive | chamaephyte | halophyte |  | All islands | 19.91342 |
| *Tolpis mariensis* Borges Silva L. & M. Moura | endemic | chamaephyte | halophyte |  | SMA | 3.463203 |
| *Tolpis maritima* Borges Silva L. & M. Moura | endemic | chamaephyte | halophyte |  | SMG | 2.597403 |
| *Trachelium caeruleum* L. | naturalised | chamaephyte | mesophyte |  | SMG, TER, FAI, FLO | 0.4329 |
| *Trifolium angustifolium* L. | naturalised | therophyte | mesophyte |  | SMA, SMG, TER, SJO, PIC, FAI | 0.4329 |
| *Trifolium campestre* Schreb. | naturalised | therophyte | mesophyte |  | All islands | 0.4329 |
| *Trifolium lappaceum* L. | naturalised | therophyte | mesophyte |  | SMA, SJO, FAI | 0.865801 |
| *Trifolium repens* L. | naturalised | hemicryptophyte | mesophyte |  | All islands | 3.463203 |
| *Tropaeolum majus* L. | naturalised | therophyte | mesophyte |  | All islands | 1.298701 |
| *Ulex europaeus* L. subsp. *europaeus* | invasive | phanerophyte | generalist |  | SMA, SMG, TER, GRA, SJO, PIC, FAI | 0.4329 |
| *Umbilicus horizontalis* DC. | native | hemicryptophyte | xerophyte |  | All islands | 3.463203 |
| *Urtica membranacea* Poir. | naturalised | therophyte | mesophyte |  | All islands | 0.4329 |
| *Vicia benghalensis* L. | naturalised | therophyte | mesophyte |  | All islands | 0.4329 |
| *Vitis vinifera* L. | naturalised | phanerophyte | mesophyte |  | All islands | 0.865801 |

List of 197 taxa gathered in 231 plots made in coastal áreas of nine Azorean islands. Abbreviations of the islands: SMA – Santa Maria; SMG – São Miguel; TER – Terceira; GRA – Graciosa; SJO – São Jorge, PIC – Pico; FAI – Faial; FLO – Flores; COR – Corvo

Supplementary Table S2 – Definitions of plant taxa categorisation, substrates and threats considered in this study.

| Category | Name | Reference | Definition |
| --- | --- | --- | --- |
| Colonisation status | Native | 12, 38 | Species that are not unique to the Azores islands. |
|  | Endemic |  | Species that are unique to the Azores islands. |
|  | Naturalised |  | Species that are well-established, with self-sustaining populations. |
|  | Invasive |  | Species that have established and spread into natural habitats, causing negative impacts on conservation. |
| Life-forms | Therophyte | 39, 40 | Annual species |
|  | Hemicryptophyte |  | Perennating buds at ground level |
|  | Chamaephyte |  | Perennating buds very close to the ground |
|  | Geophyte |  | Perennating buds below ground level |
|  | Phanerophyte |  | Perennating buds are on aerial shoots |
| Habitat ecology | Xerophyte | 35, 36, 41 | Species that thrive in dry habitats |
|  | Halophyte |  | Species that thrive in habitats exposed to salinity |
|  | Mesophyte |  | Plants that require moderate levels of moisture to thrive |
|  | Hygrophyte |  | Plants that thrive in high humidity |
|  | Generalist |  | Plants that occur and thrive in several types of habitats, having wide geographical occurrence and ecological niche. |
| Substrate | Sand | 42 | Coastal sedimentary formations that resulted from sea abrasion, with fine granulometry, usually found on beaches. |
|  | Clay |  | Material that results from basalt, trachyte, or pumice stone. |
|  | *Lapilli* |  | Volcanic gravel resulting from pyroclastic products associated with volcanic eruptions of basic magmas, very common in the Azores. |
|  | Lava flow |  | Lava formations that result from the rise of magma, which reaches the surface, during a volcanic eruption. |
|  | Boulders |  | Rock blocks of large diameters (> 0.5 m) |
|  | Rolled pebbles |  | Rounded rock fragments exposed to sea abrasion, with small – medium diameters (>0.05 m to <0.5 m), commonly found in rolled pebbled beaches. |
|  | Soil |  | Loose soil, not showing signs of compaction or of gravel content. |
|  | Rocky soil |  | Composed of a mix of gravel/rocks and soil. |
| Threats | Coastal erosion |  | Caused by the action of the sea waves on the coastline - signs of wearing and sea excavation in the cliffs, landslides, leading to terrain instability, and large fallen boulders. |
|  | Direct sea submersion |  | Areas very close to the sea level, where sea debris deposition was evident, and wherever the habitat could be directly covered by sea swell and undulation. |
|  | Storms |  | When the habitat exhibited signs of destruction caused by heavy storms (e.g., damaged plants, deposition of debris, changes in the coast line). |
|  | Agriculture |  | Presence of cultivated crops in the plot or in the immediate surrounding habitat. |
|  | Animal husbandry |  | Presence of animals, signs of trampling or grazing, deposition of faeces. |
|  | Construction work |  | Evidence of building activities occurring in or very close to the plot. |
|  | Waste disposal |  | Presence of litter or debris from human disposal. |
|  | Habitat destruction |  | A visible reduction or destruction of the available area of habitat, related with human activity. |
|  | Human infrastructure |  | Human infrastructures close to the plot. |
|  | Trampling |  | Presence of open corridors or trails originated by humans. |
|  | Invasive species |  | Presence of invasive alien plants in the plot or in the immediate surrounding areas. |

Supplementary Table S3. Climatic variables retrieved from CHELSA, including abbreviations, long names and units.

| Abbreviations | Long name | Unit |
| --- | --- | --- |
| AnnMeanTem | Mean annual air temperature | °C/10 |
| MeanDiuRange | Mean diurnal air temperature range | °C/10 |
| Isotherm | Isothermality | °C/10 |
| TemSeason | Temperature seasonality | °C/10 |
| MaxTemWarmM | Mean daily maximum air temperature of the warmest month | °C/10 |
| MinTemColdM | Mean daily minimum air temperature of the coldest month | °C/10 |
| TemAnnRange | Annual range of air temperature | °C/10 |
| MeanTemWetQ | Mean daily mean air temperatures of the wettest quarter | °C/10 |
| MeanTemDryQ | Mean daily mean air temperatures of the driest quarter | °C/10 |
| MeanTemWarmQ | Mean daily mean air temperatures of the warmest quarter | °C/10 |
| MeanTemColdQ | Mean daily mean air temperatures of the coldest quarter | °C/10 |
| AnnPrecip | Annual precipitation amount | kg m-2 |
| PrecipWetM | Precipitation amount of the wettest month | kg m-2 |
| PrecipDryM | Precipitation amount of the driest month | kg m-2 |
| PrecipSeason | Precipitation seasonality | kg m-2 |
| PrecipWetQ | Mean monthly precipitation amount of the wettest quarter | kg m-2 |
| PrecipDryQ | Mean monthly precipitation amount of the driest quarter | kg m-2 |
| PrecipWarmQ | Mean monthly precipitation amount of the warmest quarter | kg m-2 |
| PrecipColdQ | Mean monthly precipitation amount of the coldest quarter | kg m-2 |

Supplementary Table S4. Results of Kruskal-Wallis test for the colonisation status, across plot types.

| **Variable name** | **X-squared** | **df** | **p-value** |
| --- | --- | --- | --- |
| **Endemic cover** | **29.101** | **3** | **0.00528** |
| Native cover | 3.5574 | 3 | 0.3134 |
| Naturalised cover | 0.98987 | 3 | 0.8037 |
| Invasive cover | 5.155 | 3 | 0.1608 |
| **Endemic frequency** | **36.543** | **3** | **0.00192** |
| **Native frequency** | **10.12** | **3** | **0.01758** |
| Naturalised freq. | 1.3889 | 3 | 0.7081 |
| Invasive frequency | 6.6376 | 3 | 0.08439 |

Tests with significant differences are in bold. The statistical application provides the K-W statistic approximated to a chi-squared distribution, in R statistical packages.

Supplementary Table S5. Results of the Kruskal-Wallis test for the life forms, across plot types.

| **Variable name** | **X-squared** | **df** | **p-value** |
| --- | --- | --- | --- |
| **Cham cover** | **21.032** | **3** | **0.0001** |
| Hemicry cover | 7.3471 | 3 | 0.06162 |
| Phan cover | 2.6673 | 3 | 0.4458 |
| Therophyte cover | 3.2717 | 3 | 0.3516 |
| Geophyte cover | 5.8132 | 3 | 0.1211 |
| **Cham frequency** | **19.537** | **3** | **0.0002** |
| Hemicry frequency | 4.5733 | 3 | 0.2058 |
| Phan frequency | 2.5315 | 3 | 0.4696 |
| Therophyte frequency | 4.7028 | 3 | 0.1949 |
| Geophyte frequency | 5.8813 | 3 | 0.1175 |

Tests with significant differences are in bold. The statistical application provides the K-W statistic approximated to a chi-squared distribution, in R statistical packages.

Supplementary Table S6. Results of the Kruskal-Wallis test for the ecology types, across plot types.

| **Variable name** | **X-squared** | **df** | **p-value** |
| --- | --- | --- | --- |
| Xerophyte cover | 4.15 | 3 | 0.2457 |
| Mesophyte cover | 4.42 | 3 | 0.2195 |
| Hygrophyte cover | 6.1436 | 3 | 0.1048 |
| Halophyte cover | 1.5529 | 3 | 0.6701 |
| Generalist cover | 2.1066 | 3 | 0.5506 |
| Xerophyte frequency | 3.7601 | 3 | 0.2886 |
| Mesophyte frequency | 4.9715 | 3 | 0.1739 |
| Hygrophyte frequency | 5.9747 | 3 | 0.1128 |
| Halophyte frequency | 5.5583 | 3 | 0.1352 |
| Generalist frequency | 2.4958 | 3 | 0.476 |

The statistical application provides the K-W statistic approximated to a chi-squared distribution, in R statistical packages.

Supplementary Table S7. Results of Kruskal-Wallis tests conducted to community clustering and diversity, across plot types.

| **Variable name** | **X-squared** | **df** | **p-value** |
| --- | --- | --- | --- |
| **Bray-Curtis dissimilarity** | **1581.4** | **3** | **0.002** |
| **Species richness** | **23.335** | **3** | **0.02346** |
| **Shannon diversity** | **15.494** | **3** | **0.00144** |
| Evenness | 1.0909 | 3 | 0.7793 |

Tests with significant differences are in bold. The statistical application provides the K-W statistic approximated to a chi-squared distribution, in R statistical packages.

Supplementary Table S8. Results of Kruskal-Wallis and Chi-square tests conducted to environmental variables, across plot types.

| **Test** | **Variable name** | **X-squared** | **df** | **p-value** |
| --- | --- | --- | --- | --- |
| **KW** | **Elevation** | **11.495** | **3** | **0.00933** |
| KW | Exposed soil | 6.9939 | 3 | 0.07209 |
| Chi^2^ | Sand | 7.0897 | 3 | 0.069 |
| Chi^2^ | Rolled pebble | 2.067 | 3 | 0.558 |
| Chi^2^ | Lava flow | 4.5889 | 3 | 0.204 |
| Chi^2^ | *Lapilli* | 2.2616 | 3 | 0.519 |
| Chi^2^ | Boulders | 1.974 | 3 | 0.577 |
| Chi^2^ | **Soil** | **15.2649** | **3** | **0.001** |
| Chi^2^ | Field texture | 8.4191 | 6 | 0.209 |
| KW | Clay | 3.3959 | 3 | 0.3345 |
| KW | Limo | 0.29517 | 3 | 0.9609 |
| KW | Fine sand | 3.2176 | 3 | 0.3593 |
| KW | Thick sand | 1.9263 | 3 | 0.5878 |
| KW | Total N | 6.1057 | 3 | 0.1066 |
| **KW** | **Electric cond** | **12.231** | **3** | **0.006633** |
| KW | Al3+ | 1.5 | 3 | 0.6823 |
| KW | Na+ | 7.4455 | 3 | 0.05898 |
| KW | K+ | 6.9941 | 3 | 0.07209 |
| KW | Mg2+ | 7.6829 | 3 | 0.05304 |
| KW | Ca2+ | 7.7533 | 3 | 0.0514 |
| **KW** | **Olsen** | **10.637** | **3** | **0.014** |
| KW | Ext. E-R K2O | 7.7222 | 3 | 0.05212 |
| **KW** | **Ext. E-R P2O5** | **9.5629** | **3** | **0.02267** |
| KW | Org. mat. | 5.838 | 3 | 0.1198 |
| KW | KCl pH | 0.65119 | 3 | 0.8846 |
| KW | H2O pH | 0.1661 | 3 | 0.9829 |

Abbreviations: KW – Kruskal Wallis; Chi^2^ – Chi-square. Environmental parameters included elevation, exposed soil, substrate, and soil parameter variables. Tests with significant differences are in bold. The statistical application provides the K-W statistic approximated to a chi-squared distribution, in R statistical packages.

Supplementary Table S9 – Contribution of variables for the three Principal Components of the climate.

| **Variable** | **PC1** | **PC2** | **PC3** |
| --- | --- | --- | --- |
| AnnMeanTem | -0.93094 | -0.03699 | 1.606768 |
| MeanDiuRange | 0.933716 | -1.6009 | 0.026879 |
| Isotherm | 0.873954 | -1.61907 | 0.071689 |
| TemSeason | 1.512093 | -1.04172 | -0.08522 |
| MaxTemWarmM | 0.717357 | -1.54086 | 0.745052 |
| MinTemColdM | -1.33898 | 0.925317 | 0.895321 |
| TemAnnRange | 1.153271 | -1.45778 | 0.00321 |
| MeanTemWetQ | -1.74587 | -0.42514 | 0.127438 |
| MeanTemDryQ | 0.02147 | -0.76574 | 1.687564 |
| MeanTemWarmQ | -0.13706 | -0.4861 | 1.783361 |
| MeanTemColdQ | -1.08575 | 0.354732 | 1.462163 |
| AnnPrecip | 1.617951 | 0.848995 | 0.29569 |
| PrecipWetM | 1.580755 | 0.80247 | 0.375145 |
| PrecipDryM | 1.50219 | 0.639991 | 0.719697 |
| PrecipSeason | -1.15195 | 0.337604 | 0.134348 |
| PrecipWetQ | 1.564654 | 0.92857 | 0.330881 |
| PrecipDryQ | 1.606438 | 0.789715 | 0.387979 |
| PrecipWarmQ | 1.732752 | 0.470347 | 0.244815 |
| PrecipColdQ | 1.541908 | 0.854557 | 0.423388 |

Values in dark yellow boxes indicate the variables with higher positive contribution, while values in dark blue boxes indicate the variables with higher negative contribution for each Principal Component.

Supplementary Table S10. Results of Kruskal-Wallis tests across plot types, to three components of the climate.

| **Test** | **Variable name** | **Test stat.** | **df** | **p-value** |
| --- | --- | --- | --- | --- |
| **KW** | **PCA component 1** | **22.374^a^** | **3** | **<0.001** |
| Pairwise Comparisons of PlotType | Sample 1-Sample 2 | Test Statistic | Std. error | Adj. sig.^a^ |
|  | B-L | -26.174 | 33.914 | 1.000 |
|  | B-C | -58.848 | 27.949 | .211 |
|  | **B-A** | **89.449** | **28.147** | **.009** |
|  | L-C | 32.674 | 21.044 | .723 |
|  | **L-A** | **63.275** | **21.306** | **.018** |
|  | **C-A** | **30.601** | **9.215** | **.005** |
|  |  |  |  |  |
| **KW** | **PCA component 2** | **15.311^a^** | **3** | **0.002** |
| Pairwise Comparisons of PlotType | Sample 1-Sample 2 | Test stat. | Std. error | Adj. sig.^a^ |
|  | B-L | -5.114 | 33.914 | 1.000 |
|  | B-A | 65.815 | 28.147 | 0.116 |
|  | B-C | -70.655 | 27.949 | 0.069 |
|  | **L-A** | **60.701** | **21.306** | **0.026** |
|  | **L-C** | **65.541** | **21.044** | **0.011** |
|  | A-C | -4.840 | 9.215 | 1.000 |
|  |  |  |  |  |
| **KW** | **PCA component 3** | **10.432^a^** | **3** | **0.015** |
| Pairwise Comparisons of PlotType | Sample 1-Sample 2 | Test stat. | Std. error | Adj. sig.^a^ |
|  | C-A | 11.618 | 9.215 | 1.000 |
|  | C-L | -44.153 | 21.044 | 0.215 |
|  | C-B | 70.357 | 27.949 | 0.071 |
|  | A-L | -32.535 | 21.306 | 0.761 |
|  | A-B | -58.739 | 28.147 | 0.221 |
|  | L-B | 26.205 | 33.914 | 1.000 |
| The test statistic is adjusted for ties._a_ | | | | |

The three Principal components of the climate derived from a PCA applied to 19 bioclimatic variables (p≤0.05, significant differences among plot types are in bold).The statistical application provides the K-W statistic approximated to a chi-squared distribution, in R statistical packages.

Supplementary Table S11. Results of 24 binary logistic regression models tested for two target species, to explain their presence/absence.

| **Taxon** | **N** | **Model variables** | **AICc** | **pR^2^** |
| --- | --- | --- | --- | --- |
| *Azorina vidalii* | 1 | Null model. | 317.5 |  |
|  | 2 | Cover Endemics + Cover Native + Cover Naturalised + Cover Invasive + Frequency Endemics + Frequency Native + Frequency Naturalised + Frequency Invasive. | 307.666 | 0.059 |
|  | 3 | Cover Chamaephytes + Cover Phanerophytes + Cover Therophytes + Cover Hemicryptophytes + Cover Geophytes + Frequency Chamaephytes + Frequency Phanerophytes + Frequency Therophytes + Frequency Hemicryptophytes + Frequency Geophytes. | 315.321 | 0.063 |
|  | 4 | Shannon’s Entropy + Evenness. | 299.237 | 0.071 |
|  | 5 | Species Richness +Shannon’s Entropy + Evenness. | 296.479 | 0.086 |
|  | 6 | Sand + Loam + *Lapilli* + Rolled pebbles + Lava flow + Boulders + Rocky soil + Soil. | 319.882 | 0.053 |
|  | 7 | Climate component 1 + Climate component 2 + Climate component 3 + Species Richness + Frequency Endemics. | 264.234 | 0.192 |
|  | 8 | Climate component 1 + Climate component 2 + Species Richness + Frequency Endemics. | 263.561 | 0.187 |
|  | 9 | Climate component 1 + Climate component 2 + Species Richness + Frequency Endemics + Soil. | 259.545 | 0.207 |
|  | 10 | Climate component 1 + Climate component 2 + Species Richness + Frequency Endemics + Soil + Slope. | 267.299 | 0.196 |
|  | 11 | Climate component 1 + Climate component 2 + Shannon’s Entropy + Cover Endemics. | 268.432 | 0.171 |
|  | 12 | Climate component 1 + Climate component 2 + Species Richness + Cover Endemics. | 262.407 | 0.191 |
|  | 13 | Climate component 1 + Climate component 2 + Species Richness + Soil + Cover Endemics. | 258.949 | 0.209 |
|  | 14 | Climate component 1 + Climate component 2 + Species Richness + Soil + Cover Endemics + Cover Naturalised + Slope. | 260.771 | 0.224 |
|  | 15 | Climate component 1 + Climate component 2 + Shannon’s Entropy + Evenness + Soil + Frequency Endemics. | 265.647 | 0.194 |
|  | 16 | Climate component 1 + Climate component 2 + Shannon’s Entropy + Evenness + Soil + Cover Endemics + Cover Naturalised. | 262.113 | 0.212 |
|  | 17 | Climate component 1 + Climate component 2 + Shannon’s Entropy + Soil + Cover Endemics + Cover Naturalised. | 261.500 | 0.207 |
|  | 18 | Climate component 1 + Climate component 2 + Species Richness + Soil + Cover Endemics + Frequency Endemics + Cover Naturalised. | 257.526 | 0.227 |
|  | **19** | **Climate component 1 + Climate component 2 + Species Richness + Soil + Cover Endemics + Cover Naturalised.** | **256.953** | **0.222** |
| *Lotus azoricus* | 20 | Null model | 123.4 |  |
|  | 21 | Climate component 1 + Climate component 2 + Climate component 3 + Species Richness + Frequency Endemics. | 83.764 | 0.413 |
|  | 22 | Climate component 1 + Species Richness + Frequency Endemics + Boulders + *Lapilli* + Lava flow + Soil. | 89.870 | 0.398 |
|  | 23 | Climate component 1 + Species Richness + Frequency Endemics. | 84.747 | 0.370 |
|  | **24** | **Climate component 1** **+ Shannon’s Entropy + Frequency Endemics.** | **81.545** | **0.396** |

Results include AICc and pR^2^ values. Each model was built using different combinations of variables, including plant categorisation, plant diversity indexes and environmental variables. Only variables with significant regression coefficients (<0.001 ‘***’ 0.001 ‘**’ 0.01 ‘*’) were selected for the final models. The best models for each species are represented in bold. For *A. vidalii*, the best model showed a significant contribution of climate components 1 and 2, higher species richness, presence of soil substrates, and greater endemic and naturalised cover. For *L. azoricus*, the best model showed a significant contribution of climate component 1, Shannon’s entropy and higher frequency of endemic species.

Supplementary Table S12. Results of Chi-square tests conducted to the threats, across plot types.

| **Variable name** | **X-squared** | **df** | **p-value** |
| --- | --- | --- | --- |
| Reduction/destruction of habitat | 0.98899 | 3 | 0.8039 |
| Waste disposal | 4.880 | 3 | 0.1807 |
| Coastal erosion | 4.661 | 3 | 0.198 |
| Invasive species | 2.0576 | 3 | 0.560 |
| **Direct sea submersion** | **13.680** | **3** | **0.003** |
| **Animal husbandry** | **8.2327** | **3** | **0.041** |
| Trampling | 1.7598 | 3 | 0.6237 |
| Human infrastructure | 4.759 | 3 | 0.190 |
| Storms | 2.993 | 3 | 0.3926 |

Tests with significant differences are in bold.

Supplementary Table S13. Percentages of plots from each target taxa located within the different protected areas frameworks.

| **Taxon** | **Island** | **INP** | | **Natura 2000** | | **Total** |
| --- | --- | --- | --- | --- | --- | --- |
|  |  | **PLA** | **PAMSH** | **SPZ** | **SCZ** |  |
| *Azorina vidalii* | **SMA** | 31.58 | 47.37 | 0.00 | 36.84 | 78.95 |
|  | **SMG** | 0.00 | 20.00 | 0.00 | 0.00 | 20.00 |
|  | **TER** | 0.00 | 14.29 | 0.00 | 14.29 | 14.29 |
|  | **GRA** | 0.00 | 0.00 | 0.00 | 0.00 | 0.00 |
|  | **SJO** | 35.71 | 21.43 | 7.14 | 28.57 | 57.14 |
|  | **PIC** | 12.50 | 6.25 | 6.25 | 12.50 | 25.00 |
|  | **FAI** | 50.00 | 0.00 | 25.00 | 50.00 | 50.00 |
|  | **FLO** | 7.14 | 50.00 | 7.14 | 35.71 | 57.14 |
|  | **COR** | 0.00 | 0.00 | 0.00 | 0.00 | 0.00 |
|  | **Total** | *16.16* | *24.24* | *4.04* | *21.21* | **41.41** |
| *Lotus azoricus* | **SMA** | 0.00 | 100.00 | 27.27 | 72.73 | 100.00 |
|  | **SJO** | 100.00 | 0.00 | 0.00 | 100.00 | 100.00 |
|  | **PIC** | 40.00 | 0.00 | 40.00 | 40.00 | 40.00 |
|  | **Total** | *17.65* | *64.71* | *29.41* | *64.71* | **82.35** |

Short names meanings as follows: (i) areas belonging to Island Natural Parks (INP): PLA – Protected Landscape Areas; PAMSH – Protected Areas for Management of Species and Habitats; ii) Natura 200 Network: SCZ – Special Conservation Zones; SPZ - Special Protection Zones. Abbreviations of the islands: SMA – Santa Maria; SMG – São Miguel; TER – Terceira; GRA – Graciosa; SJO – São Jorge, PIC – Pico; FAI – Faial; FLO – Flores; COR – Corvo. At the end of each line, we provide the total percentage of plots within protected land for each taxon and island. Italicised values represent the total percentage of plots within each protected area typology, for each taxon. Values in bold represent the total percentage of plots within protected areas per taxon.

Supplementary Table S14. Area of occupancy estimated with GeoCAT, in km^2^, per island, for two target species.

| Island | Area of occupancy in km^2^ (using 4 km^2^ parcels) | | Area of occupancy in km^2^ (using 1 km^2^ parcels) | |
| --- | --- | --- | --- | --- |
|  | ***A. vidalii*** | ***L. azoricus*** | ***A. vidalii*** | ***L. azoricus*** |
| SMA | 14 | 6 | 9 | 3 |
| SMG | 28 |  | 14 |  |
| TER | 10 |  | 5 |  |
| GRA | 2 |  | 1 |  |
| SJO | 22 | 2 | 12 | 1 |
| PIC | 24 | 8 | 15 | 5 |
| FAI | 8 |  | 4 |  |
| FLO | 18 |  | 12 |  |
| COR | 6 |  | 3 |  |
| Total | 132 | 16 | 75 | 9 |

Abbreviations of the islands: SMA – Santa Maria; SMG – São Miguel; TER – Terceira; GRA – Graciosa; SJO – São Jorge, PIC – Pico; FAI – Faial; FLO – Flores; COR – Corvo.

Supplementary Table S15. Estimated numbers of mature individuals of the target taxa, per island and in total.

| Island | Number of mature individuals | |
| --- | --- | --- |
|  | ***A. vidalii*** | ***L. azoricus*** |
| SMA | 660 | 1310 |
| SMG | 1120 |  |
| TER | 600 |  |
| GRA | 250 |  |
| SJO | 3070 | 10 |
| PIC | 2800 | 200 |
| FAI | 720 |  |
| FLO | 830 |  |
| COR | 1200 |  |
| Total | 11250 | 1520 |

Abbreviations of the islands: SMA – Santa Maria; SMG – São Miguel; TER – Terceira; GRA – Graciosa; SJO – São Jorge, PIC – Pico; FAI – Faial; FLO – Flores; COR – Corvo.
